# Systemic effects of the COVID pandemic on rural black American men’s interpersonal relationships: A phenomenological examination

**DOI:** 10.1371/journal.pone.0297876

**Published:** 2024-04-17

**Authors:** Michael G. Curtis, Elizabeth Wieling, Chalandra Bryant, Rosalyn Denise Campbell, Steven M. Kogan

**Affiliations:** 1 Department of Global Health Rollins School of Public Health, Emory University, Atlanta, Georgia, United States of America; 2 Department of Human Development and Family Studies, University of Georgia, Athens, Georgia, United States of America; 3 Department of Family Social Science, University of Minnesota, Minneapolis, Minnesota, United States of America; 4 Independent Scholar, Athens, Georgia, United States of America; Nazarbayev University, KAZAKHSTAN

## Abstract

The COVID-19 pandemic was a socionatural disaster that unprecedentedly disrupted the daily lives of individuals, families, and communities. Prior research indicates that Black American men living in rural contexts, particularly in Southern parts of the United States of America, were disproportionately affected by the psychological and economic effects of the pandemic. Despite these disparities, few studies have examined the pandemic’s impact on rural Black American men’s social networks. This study aimed to explore the effects of the COVID-19 pandemic on rural Black American men’s interpersonal relationships. Informed by the principles of critical ethnography and guided by van Manen’s hermeneutic phenomenology, seventeen men were interviewed using a semi-structured interview protocol. Interviews were transcribed and then analyzed using an iterative thematic reduction process consistent with van Manen’s approach. Four themes were generated: Familial Reorganization, Adaptive Fatherhood, Rona Romance, and Essential Community. Participants recounted how the pandemic motivated them to improve their relationships with family members and children but contributed additional stress to their romantic relationships. Participants further recounted how their friendships were the least impacted as they were willing to make exceptions to their normal protective protocols to socialize with close friends. Participants also noted feeling disconnected from their wider community because they could not attend church even though their religious beliefs remained unchanged. Findings highlight the need for scholars, clinicians, and policymakers to consider men’s relational health when developing and implementing pandemic recovery efforts, as it can significantly influence their ability to recuperate mentally and physically. Future research should be dedicated to (1) investigating the effects of the COVID-19 pandemic on fathers, as prior research has nearly exclusively focused on mothers’ experiences and (2) delineating protective effects of rural Black American men’s involvement in the Black Church from their individual spiritualities to gain a more comprehensive understanding of the influence of contextual crisis on their long-term health and wellbeing.

## Introduction

Epidemiological evidence indicates that Black Americans were more likely to be diagnosed, hospitalized, and die from the COVID-19 virus [1–3]. In Georgia, COVID-19-related health disparities quickly emerged in the first seven weeks of community spread across the state [4]. Although urban areas such as Metropolitan Atlanta exhibited the largest number of confirmed cases, less populated southwestern counties, such as Dougherty, exhibited the highest biweekly increase in incidence and mortality rates per 100,000 population [4]. Evidence indicates that counties with the highest mortality rates were also more likely to have a higher proportion of Black American individuals, older than 60 years, living with yearly incomes less than $20,000, and living in rural areas [5]. Rural counties were also more likely to have fewer intensive care unit beds per 100,000 people and fewer primary care physicians per 10,000 people, indicating a possible deficiency in access to medical care and the ability to handle disease outbreaks [4]. A possible explanation for the overrepresentation of Black Americans in confirmed COVID-19 morbidity and mortality cases may be related to preexisting social, economic, and health inequalities tied to race, class, and access to the healthcare system.

### Black American men’s health in times of crisis

Black Americans, particularly in the rural South, are often suspicious of the health system, with a legacy of abuses such as the 1932–1972 Tuskegee syphilis study in rural Alabama, where Black American participants were allowed to die untreated to observe the natural history of untreated syphilis [6, 7]. Unfortunately, the UNITED STATES OF AMERICA health system has repeatedly been shown to offer inferior care to Black Americans with the same conditions and insurance as White American patients, indicating the presence of persistent racial bias in health care [8].

Among Black American men, white American-patriarchal social structures sponsor social and economic actions that have marginalized their experiences and nullified some of the masculine privileges afforded to them by the contemporary global zeitgeist [9]. The inability to attain masculine roles has often kept Black men from materializing even the most fundamental aspects of patriarchal privilege and power [9]. Specifically, the development of Black American masculinity occurs within a precarious context of overt and covert discrimination and structural racism that have denied Black American males more familiar routes to public masculinity, including decent wage-paying jobs, opportunities to accrue status or personal power, and the traditional patriarchal role as head of the family, disciplinarian, and primary breadwinner [10].

Faced with these barriers, many Black American men have constructed alternative forms of masculinity based on what was available in their everyday interactions, more suited to their narrower horizons and not dependent on the dominant culture [11]. For example, the rigid codes of manhood adopted by many Black American men dictate that they should man up by "toughing out" pain, injury, and illness and avoid complaining or seeking help from others [12]. In turn, Black American men may risk their physical health and well-being rather than be associated with traits they or others may perceive as feminine [12]. As a result, Black men may avoid seeking medical help until their bodies are in crisis from treatable, and in some cases, even preventable, illnesses such as COVID-19 [13]. The persistence of barriers to achieving and expressing more adaptive forms of manhood has generally resulted in significant social disadvantages for Black American males. Such disadvantages have contributed to myriad disparities that can magnify the effects of the COVID-19 pandemic on Black American men. To date, research on the effects of the COVID-19 pandemic among Black American men has been dominated by literature articulating its physical and mental health effects. While essential for constructing pandemic-recovery interventions, these investigations often decontextualize Black American men from the interpersonal systems that help shape their lived experiences.

### Importance of interpersonal connections

Extensive research has demonstrated an association between interpersonal relationships and major life stressors [14, 15]. Prior research indicates that relationships that instill feelings of safety can facilitate self-disclosure, emotional resiliency, coping strategies, and additional social support resources to protect against major life stressors’ physical and mental health burden [16, 17]. In contrast, major life stressors can tax individuals’ abilities to sustain positive interpersonal relationships [18]. For instance, school closures and disruption of family care seriously disrupt regular domestic practices resulting in increased relational distress and instability.

Over the last few years, the COVID-19 pandemic has been a considerable socioecological stressor [19, 20]. Findings from previous research indicate that Black Americans were among the most impacted racial/ethnic groups [21]. Evidence suggests that the pandemic uniquely impacted Black American men living in the rural South due to (a) being overrepresented in industries that require face-to-face interactions with the public, (b) living in multigenerational housing, and (c) having limited access to preventative and emergency health care [22–24].

Very few studies have examined the association between interpersonal relationships and the COVID-19 pandemic; those that do have yielded conflicting results. For instance, Chakraborty et al. demonstrated that COVID-19 pandemic-related distress was associated with increased relational distress, which, in turn, was associated with partners more frequently (1) pointing out each other’s faults, (2) attempting to engage one another for attention, affirmation, or affection, and (3) engaging in relational violence [25]. Conversely, research indicates that the COVID-19 pandemic evidenced several positive effects on intimate relationships, including developing deeper levels of understanding between partners, as well as increased levels of sense of trust, affection, care, emotional support, and shared childcare responsibilities [25]. Other studies demonstrated similar results and showed strong associations between pandemic-related stress and increased levels of intimate relational distress, relational uncertainty, domestic violence, and decreased levels of perceived intimate relationship quality [26–29]. Prior literature in this area has been limited in two ways. First, these studies only focused on romantic relationships and did not address other salient interpersonal relationships, such as the parent-child relationship and friendships. Second, the samples used in these studies were either international or consisted of primarily White American and female-identified participants limiting their generalizability. These data highlight the need for targeted research examining how the COVID-19 pandemic influenced the interpersonal relationships of Black American men.

### Paradigmatic orientation

#### Critical post-modernism

The aims of the current study are paradigmatically aligned with the tenets of critical post-modernism. Critical post-modernism can be characterized by (a) the crisis of representation, which rejects the idea that a researcher’s work is an objective depiction of a stable phenomenon and (b) the tempering of social constructionists’ beliefs regarding reality with the awareness of how complicated, interconnected social, structural, cultural, political, economic, and material realities contribute to the unequal division of labor, resources, power, and agency [30]. The unequal division of labor, resources, power, and agency greatly influences what individuals deem as normative, how they make meaning of their thoughts, feelings, and behaviors, how they ascribe meaning to specific experiences, and how they idealize or pathologize themselves and others [31]. Through the use of a critical post-modern paradigm we sought to highlight and privilege the voices of those at the margins of dominant discourses that have been silenced and largely excluded from historical narratives [32].

#### Social constructionism

Through the lens of critical post-modernism, we have integrated tenets of social constructionism, a theory of knowledge centered around the notion that the interpretation of lived experiences is conducted in coordination with others rather than separately within each individual [33]. Instead of there being one singular reality with universal truths that can be applied to all people at all times, social constructionists assert that each person constructs a personal reality that is a subjective, biased, and abridged mental image of the world [34]. We posit that reality is not some objective truth waiting to be uncovered through positivist scientific inquiry but constructed through various shared realities. As such, we further posit that language serves as a communicative tool that does not mirror reality but rather constitutes it.

Within social constructionism, we adopted the position that knowledge generation is primarily a relational process [35]. Knowledge generation is deeply connected to the process of interpretation and the collaborative, interpersonal construction of reality [36]. In alignment with this perspective, we actively turned away from the individual to the social by shifting my understanding of knowledge from an individual cognitive construction to a communal one, from language as representational to language as a dynamic social process, and from the notion of a person as a bounded self to the notion of a person as a relational system embedded within out relational systems [36]. For example, in examining the lived experiences of rural Black American men, we believed it was imperative that the interpretation of their thoughts, feelings, and behaviors be done in the context of years of systemic racial and cultural oppression where their Blackness and maleness was under constant challenge and scrutiny, and contemporary forms of marginalization that restricts their access to biological, psychological, and social health and socioeconomic mobility.

### Guiding theoretical frameworks

#### Intersectionality

Intersectionality articulates how interdependent systems of discrimination and disadvantage influence the lived experience of individuals, families, and communities [37]. Although intersectionality was originally developed as a framework to explain Black women’s experiences, it also applies to Black American men [38, 39]. Like Black women, Black American men’s identities straddle the intersection of several marginalized identities. For instance, prior research suggests that Black American men are unique in how they exhibit manhood [40]. This uniqueness emerges from their shared experiences with slavery, racial segregation, and race relations that hold special meaning for members of African descent. Historically, society has created an ideal image of Black American men without acknowledging their experiences with oppression and racism. By viewing Black masculinity through this intersectional lens, Black masculinity blends traditional White American masculine standards with Black American cultural standards. This uniqueness complicates their ability to live up to these standards, and it becomes further complicated by the added effects of racism and discrimination [41].

A strength of intersectionality, relative to other critical social theories, is that it challenges the idea of a single social category (such as race or gender) being the primary dimension of inequity; instead, it asserts that multiple dimensions can and do shape social inequality [42]. From this perspective, the gendered, racialized, and economic factors that shape Black American men’s health and wellbeing cannot be understood independently but are inextricably intertwined and experienced simultaneously along with other social determinants of health. Only through an intersectional lens can these processes be adequately documented and understood [43]. Intersectionality is relevant to this research by providing a critical lens through which we sought to gain an in-depth and nuanced understanding of how rural Black American men’s unique social location influences their experience of the COVID-19 pandemic.

#### Family systems theory

Family systems theory posits that the lives of individuals, couples, and families exist as cohesive conglomerations of interrelated parts, wherein each part is delineated by its own spatial and temporal boundaries [44]. These parts are referred to as systems, which implies a stable configuration that is maintained by the coordination of various components through an internal network and the characteristics of the larger system of which it is a component [44]. Therefore, systems simultaneously exist as wholes in and of themselves as well as components of larger systems, which are constantly influencing and being influenced by one another [45]. Scholars have come to understand how one system influences another by investigating power processes [46]. Such processes govern systems’ structure, purpose, and nature [46]. For instance, individuals are often characterized as the least powerful within power processes of larger societal systems; however, an individual’s role within society may significantly alter their degree of influence on other systems (e.g., breadwinners, primary caregivers). Each system requires its own characterization and investigation within intersystem interactions [44].

Family systems theory provides an explanatory framework for understanding how individual- and environmental-level changes can influence family or interpersonal systems’ functionality and characteristics. For example, changes in employment may influence Black American men’s mental health, which may directly affect their ability to communicate empathically with their partners, children, and family members, which in turn may have significant ramifications on their family’s functioning and stability. Public health scholars have predicted that the COVID-19 pandemic will indirectly affect rural Black American men’s intrapersonal and interpersonal health by disrupting formal and informal social support networks [47]. In accordance, I utilized systems theory to understand better how the COVID-19 pandemic can have proximal implications on rural Black American men’s interpersonal relationships.

#### The current study

The current study aimed to explore the effects of the COVID-19 pandemic on rural Black American men’s interpersonal relationships. Findings may be used to (a) expand scholars’ understanding of how socionatural disasters relational impact highly marginalized and disenfranchised communities, such as rural Black American men; (b) inform pandemic response and recovery efforts within highly marginalized and disenfranchised communities; and (c) inform the development of productive and culturally responsive clinical practices and interventions.

## Materials and methods

### Research positionality

I, the first author, am Queer, Black American man who grew up in a low-resource environment; I am the first person in my family to attend college. Although my parents were extremely hard workers, our family experienced frequent bouts of food and housing insecurity. Growing up in this context has made me keenly sensitive to the systems of oppression that impact the health and wellbeing of Black American men. As such, I was, and remain, deeply connected to the men’s lived experiences highlighted in this study. This research project was completed as a component of my doctoral dissertation; [second author] and [last author] served as my major professors, and [third author] and [fourth author] served as mentors and committee members. I, and my coauthors, approached this study with great humility and reverence for the communities highlighted in this work.

### Hermeneutic phenomenological qualitative approach

This study was guided by van Manen’s approach to hermeneutic phenomenology [48]. Phenomenology is the study of (a) phenomena as they manifest in people’s lived experience, (b) how people perceive and understand phenomena, and (c) the meaning phenomena have in people’s subjective lived experience [48]. The term *lived experience* refers to people’s encounters with everything within their lifeworld—i. e. the world as it appears to them and is salient to them [49]. van Manen’s unique approach to hermeneutic phenomenology challenges researchers to acknowledge and leverage their own social, historical, and cultural experiences to enhance their understanding of how individuals make sense of their lived experiences [48]. This approach was best suited for the current research study due to the first author’s insider status as a member of the Black American community and the study’s aim to examine and document rural Black men’s experience of the evolving COVID-19 pandemic.

#### Principles of a critical ethnography approach

Madison’s critical ethnography approach was a second methodological framework [50]. Critical ethnography acknowledges that power differentials and oppression operate at all levels of human interaction and that oppressive organizational structures greatly influence rural Black American men’s lifeworlds [51]. Two key principles underscore this approach. First is the notion that every aspect of one’s lifeworld can only make sense when contextualized; second, individuals are intensely knowledgeable, consciously or subconsciously, about their lifeworlds and are, therefore, reliable informants [52]. This approach emphasizes interpreting culture so that interpretations promote cultural change. Critical interpretations examine participants’ lived experiences through the lens of power, privilege, and authority by uncovering unfair and unjust systems and revealing whose voices and personal stories are “heard” and whose voices are “silenced” [53]. The integration of critical ethnography principles into a hermeneutic phenomenological framework can (a) counter the production of surface representations of participant’s lived experiences, (b) disrupt the perpetuation of the status quo, and (c) unearth taken-for-granted assumptions within the researcher’s and participant’s lifeworlds [54].

### Participants

The sample was recruited from active African American Men’s Project (AMP) participants. AMP is an ongoing longitudinal study that follows a cohort of 504 young adult Black American men. Men were recruited from 11 contiguous counties in rural Georgia in the United States of America, representing rural poverty areas in the southeastern United States [55]. Initial eligibility criteria included: self-identification as Black or African American, residence in the sampling area, cisgender male, and age of 19–22 years. Participants participated in yearly follow-up surveys. The COVID-19 pandemic occurred after Wave 4 of quantitative data collection was completed (i.e. January 2020). The first wave of interviews was conducted from April 2020 to December 2020, while the second wave was conducted from September 2021 to October 2021. Seventeen men agreed to be interviewed for this study, with eight agreeing to be interviewed again a year later. Demographic data taken from Wave 4 of quantitative data collection is provided in Table 1. During the first wave of interviews, participants were approximately 27 years old (M = 27.20, SD = 1.23, Range = 3.35). Additionally, most participants were never married (94.10%), living with a romantic partner (35.3%), had earned at least a bachelor’s degree (35.30%), were employed (88.20%), and had one or more children (47.10%). In-depth individual descriptions of each participant are provided in S1 Table. Prominent details such as participant’s name and the location of certain events have been withheld or changed to protect their identities.

**Table 1:** Participant demographics.

Characteristic	*N* (17)	%
**What is your current marital status?**		
** Never Married**	16	94.1
** Missing**	1	5.9
**Do you currently have a main romantic partner?**		
** Yes**	8	47.1
** No**	8	47.1
** Missing**	1	5.9
**Do you and your main romantic partner live together?**		
** Yes**	3	17.6
** Yes, most of the time**	1	5.9
** Some of the time**	3	17.6
** No**	1	5.9
** Not In a Relationship/Missing**	9	52.9
**What are your current living arrangements?**		
** I live with my spouse or romantic partner**	6	35.3
** I live alone or with my children**	1	5.9
** I live with the family I grew up with**	3	17.6
** I live with other relatives**	3	17.6
** I live with friends or roommates my age**	3	17.6
** Missing**	1	5.9
**What is the highest level of education you have completed?**		
** High School Diploma**	5	29.4
** One year of college or trade school**	4	23.5
** Bachelor’s Degree**	6	35.3
** Master’s Degree**	1	5.9
** Missing**	1	5.9
**Are you currently enrolled in school or any type of educational program?**		
** No**	14	82.4
** Yes, in a 4-year college or university**	1	5.9
** Yes, in graduate school**	1	5.9
** Missing**	1	5.9
**Currently Employed**		
** No**	1	5.9
** Yes**	15	88.2
** Missing**	1	5.9
**Do you receive health insurance benefits through your work?**		
** No**	3	17.6
** Yes**	8	47.1
** Missing**	6	35.3
**Do you have retirement benefits taken from your paycheck?**		
** No**	4	23.5
** Yes**	7	41.2
** Missing**	6	35.3
**How many children do you have?**		
** 0**	8	47.1
** 1**	4	23.5
** 2**	1	5.9
** 3**	1	5.9
** 4**	1	5.9
** 7**	1	5.9
** Missing**	1	5.9

### Interview design

Two waves of qualitative interviews–wave 1 included all 17 men and wave 2 included 8 of the original 17—were used to gather data. In the first wave, questions were designed to explore the influence of the COVID-19 pandemic on rural Black American men’s lived experience, including their biological, psychological, and social functioning, relational health, and socioeconomic mobility (See S1 File). Participants were not prompted to speak on another subject until they *ran out of steam* to produce data that would have otherwise been missed [56]. Participants were asked to clarify and expand on topics that needed further elaboration. The second interview, which took place approximately one year later, centered on member checking emerging topics, ideas, and patterns of meaning that repeatedly arose within and across interviews and new topical areas (e.g., attitudes towards vaccines, ongoing individual, and family wellbeing) were explored. The first author solely conducted both waves of interviews.

### Procedure

Each potential participant received a phone call inviting them to participate in this study. During this initial call, the first author explained the study’s purpose and the participation requirements. At that time, any questions the study participants posed were answered. Once participants agreed to participate in this study, they were provided a link, either over the phone or via phone text message, to an online webpage where they could read and sign the consent form. After the written consent process was completed, the first author contacted participants via phone call or text message to explain the study’s aims and identify their preferred videoconferencing platform (e.g., Facetime or Zoom) for communication and choose a date and time for their interview. Regardless of platform, each interview was only audio-recorded using audio recording software not integrated into the videoconferencing platform. After each interview, audio files are stored on a secure HIPAA-compliant OneDrive server. The second interview took place approximately one year after the first interview. The same process, except for the written consent, was used to schedule the second interview. Each interview was expected to last approximately 60–90 minutes. Once the interview was completed, the first author sent participants a self-addressed, stamped envelope and receipt for a $100.00 check. This project was approved by the University of Georgia’s Institutional Review Board (IRB; #PROJECT00002219).

#### Confidentiality

Participants were instructed that they may skip any part of the study that they felt uncomfortable discussing without penalty. This was explained within the consent form and at the beginning of each interview. No information collected from interviews was shared with individuals outside the research team. The audio recordings and transcripts were stored in a safe place, encrypted and behind two locks, away from public access, and only accessed by the research team. Prominent details such as participants’ names and the location of certain events have been withheld or changed to protect their identities. Pseudonyms were used to protect participants’ identities.

#### Data analysis

Interview transcripts were analyzed using iterative thematic analysis in NVIVO, a qualitative data management and analytic tool that helps qualitative researchers organize, analyze, and find insights in qualitative data [48]. A thematic analysis aims to identify, analyze, and interpret patterns of meaning (or "themes") within a given dataset. van Manen’s levels of thematic analysis were conducted at three levels: holistic, detailed, and selective [48]. First, at the end of each wave of data collection, holistic summaries (i.e., belief narratives that recounted the major points) were created for each interview. Next, transcripts underwent a process of inductive data analysis, also called detailed coding, which involved assigning a label to key sections of interviews using a word or short phrase–*a code*–provided by participants [48]. This analysis level aimed to code as many meaningful segments as possible; thus, each interview was read and coded at least twice. Some codes were repetitive and clustered under the same code during this process.

Next, codes underwent a process of deductive analysis, also called selective coding, that focused on clustering similar codes into themes, i.e., abstract concepts that bring meaning and identity to a recurrent phenomenon and its various manifestations [48]. Emerging themes were identified via interpretive analysis wherein codes were distilled into essential facets of participants’ lived experiences. The process of theme generation focused on identifying how each participant came to make sense of their lived experiences. Across all analysis levels, themes and codes were continuously evaluated to prevent any loss of meaningful information by assessing the relevance of these concepts to participants’ information with modifications or revisions of codes and themes to be applied as necessary.

Comparisons of data against data across and between (a) participants, (b) codes, and (c) themes were performed throughout the data analysis process. Codes and themes also underwent sequential comparisons whereby information from prior interviews was compared against information provided by subsequent interviews across and between participants [57]. This iterative process continued until thematic saturation was reached. Thematic saturation refers to a point at which observing more data is not likely to lead to the discovery of more thematic information related to the research questions [58]. For this study, thematic saturation was achieved when—in wave one of data collection and analysis—no additions or modifications to existing codes and themes in the codebook were warranted. This approach acknowledges that the researcher plays an active role in forming an understanding of the participants’ experiences; that is, the researcher will bring their own experiences and expertise to the analysis, and this must be reflected upon by keeping a reflexive attitude and maintaining a keen awareness of the role any researcher biases and preconceptions within the analytic process.

#### Trustworthiness

Qualitative trustworthiness refers to the accuracy of a research study, data, and findings to reflect participants’ lived experiences [57]. Guba and Lincoln’s tenets of qualitative trustworthiness were used to evaluate this study’s implementation and data analysis: credibility, transferability, dependability, and confirmability [59].

#### Credibility

Credibility refers to the confidence that can be placed in the *truth* of the research findings [59]. The credibility of this study was established using: (a) analyst triangulation with coauthors and (b) memo writing [60, 61]. Memoing was used to document research decision-making processes and track emerging theorizing regarding (a) researcher’s interpretation of emergent findings, (b) the construction of concepts, categories, properties, and themes, (c) the rationale used to operationalize analytic constructions, and (d) the relationships between these analytic constructions [62].

#### Transferability

Transferability refers to the degree to which qualitative research results can be generalized or transferred to other contexts or settings [59]. The transferability of this study’s results was demonstrated by presenting thick descriptions via providing detailed quotes from participants in the results section to illustrate their experiences and directly from their point of view [61].

#### Dependability

Dependability is demonstrated by using reliable methodologies that are consistent and can be replicated [59]. External audits involve a secondary researcher examining the research process and congruences between research methodology and resulting data collection. Within the context of this study, [second author] and [last author] authors performed external audits throughout the research process.

#### Confirmability

Confirmability refers to the extent to which the study’s results represent participants’ lived experiences instead of researcher bias and motivations [59]. Confirmability auditing is a form of targeted auditing that focuses on thoroughly examining research data and its interpretation [59]. Within this study, confirmability auditing was conducted by [second author] and [last author].

## Results

Interpersonal relationships were essential to men’s experiences during the COVID-19 pandemic. All participants discussed how the pandemic impacted their relationships with parents, siblings, children, intimate partners, and friends. For some participants, these relationships grew stronger as they recounted increasing the quality and frequency of their communication with loved ones. Others found it more difficult to navigate and maintain healthy interpersonal relationships when faced with the additional stress of the pandemic. Four essential themes emerged and related sub-themes: (1) Familial Reorganization, (2) Adaptive Fatherhood, (3) Rona Romance, and (4) Essential Community.

### Familial Reorganization

For all participants, the COVID-19 pandemic allowed them to slow down their daily activity levels. This slowdown allowed participants to reflect on the status of their familial relationships deeply. Consequently, several participants reported that this process of reflection encouraged them to address lingering relational issues to improve their quality of life and relational health. Two additional subthemes emerged: Breath of Fresh Air and Just More Bad News.

#### Breath of Fresh Air

Jackson (28-years-old) discussed how reductions in daily responsibilities and activity levels allowed him to reconnect with family members:

I mean, I think it (the COVID pandemic) actually helped me a lot. Because I’m not, I used to be real speedy, speedy, speedy, run here, run there, run there. I was busy. When COVID came, it weren’t that busy, there were way less things to do and places to go, and all that. Everything was shut down, so I spent a lot more time with my family. A lot more time with my family, and I appreciate it.

Indeed, many men discussed strengthening family bonds and reconnecting with distant family members during the pandemic out of fear that they would lose loved ones or family members without communicating with them. In our first interview, Eric (27-years-old) expounded upon how important it was for him to reconnect with family members in this way:

I learned that you know the virus. It’ll make you get closer to your family. Because a lot of people were losing family members, you know? And people were losing people that they didn’t normally talk to on the regular, and you know, just losing people. And I felt that the virus, it made a lot of people, I’d say, reconnect with their family. Get back good with their family. Cherishing their families in the moment that they still have with their family members. Because you never know. With the virus, you never know what’s going to happen. You never know who you’re going to lose.

Participants often attributed their reconnection with family members to their ability to slow down their daily activity and reflect upon the relationships that are important to them. Jackson (28-years-old) posited that this additional time allowed him to grow closer to his children:

I think my experience, my main experience with COVID, is just like I really got closer to my household. My kids are out of school so long that I just get so many more hours with them than normal. I had to teach them, because it was like, it’s bad but it’s good. It was really a good experience. Not necessarily good, but the best out of it, like it was better for building households if you had the structure to do so. You know what I mean? Because it took everybody else away for a while, you know what I mean? It was just you and your family, and I appreciated that.

The theme of reconnecting to family persisted well into the second wave of interviews as men felt more comfortable traveling and physically reconnecting with family members. For instance, Tyrone (28-years-old) describes traveling to see family in a different state, after not seeing them for over a year, as feeling like a breath of fresh air:

It’s like a breath of fresh air. If you’ve ever been in a pool and you go down, and you just try to hold your breath as long as you can, and then you come up, and you take that deep breath. It’s kind of like that. It’s just a breath of fresh air. I mean, to see them smile, to see the hugs, to get the home-cooked meal that you’re missing, and just to be around the people that love you the most, as I’ve said, it’s like a breath of fresh air.

#### Just More Bad News

Stronger familial relationships due to the effects of the pandemic were not homogenous, as some men recounted feeling a growing distance within their family systems. This distance was associated with a decrease in physical interaction due to a fear of endangering older members of the family system, who were often peacekeepers:

We’re just becoming more distant, actually…Because Grandma’s so old, so they don’t want to take no chances, so nobody gets to go around. So nobody gets to even go around each other. You ever seen the movie Soul Food? You know how when Big Mama die, you know nobody was going to come together no more? It’s one of those situations. When granny is gone, then everything is. Nobody really gets along in the family, but grandma makes everybody get along (Jackson, 28-years-old).

This distance was also related to self-preservation. Some men chose to limit their communication with family members out of fear that the information they received would be more bad news. Ben (27-years-old), who acquired COVID-19twice and reported struggling with his mental health due to these experiences, limited his interactions with others to protect his mental health:

I’m just kind of the secluded. I’d kind of isolated myself. I know. I just like been chilling by myself. Not really. I’ve been kind of out of touch with them lately. No particular reason. It’s just like, I just like been like, uh, like I’m going to call my sister, and I don’t call or something like that. I talked to her last night without, you know, she was just telling me no that one of my aunts, you know, she was really sick, and she wasn’t doing too well, so I never followed call her back. It was like, oh, more bad news. It’s like, I’ll talk to you later.

#### Adaptive Fatherhood

Men who identified as fathers described how they adjusted their parenting styles, values, and behaviors to be more responsive to the needs of their children. They attributed this change to the pandemic disrupting their family systems’ normative habits and behavior. Two additional subthemes emerged: Mentally Present and Renovating Parenting.

#### Mentally Present

Fathers reported that greater fatherhood involvement was a salient positive outcome of the pandemic. All fathers discussed the importance of having more time with their children and being mentally present during those times. Elias (27-years-old) explained how time off from work allowed him to be more present with his children:

It’s given me the time to make sure I’m able to show them and teach them these things now, instead of just being in a restaurant all the time, trying to make money, trying to get to work on time. It’s a lot going on, now I can just work in the yard and if they want, they can be present with me. They get in the way a little bit, but hey, it beats not seeing them, being on the clock.

#### Renovating Parenting

These moments of mental presence allowed some fathers to redefine their relationship with their children. Jackson (28-years-old) discussed how the additional time he spent with his children gave him a new perspective on the relationship he would like to have with them:

I’m nicer now, though. Yeah. I learned how to be…Definitely let them be more kids, you know what I mean? I used to be a little hard on them, do this, do that, find them chores, and stuff like that. But now, you know what I’m saying? I let them talk, and they ask questions, do whatever, do whatever. You know? Try and let them just be individuals because I realized that you can’t make people do stuff that they don’t want to do.

During these times of connection, some fathers reported having to change their behaviors to be adaptive to their children’s current environmental limitations. For example, despite Bernard’s (26-year-old) value of limiting his children’s access to electronic devices, he adapted to their new context of not being able to play outside or with friends by allowing them to play more video games or watch tv for longer periods:

I love video games, but I have a 11-year-old and a seven-year-old and a five-year-old, at that age I wasn’t really playing video games. I was outside and you couldn’t keep me in the house unless I had a game, but outside would be like my go-to and then the game would be back up. For the kids it’s kind of, they like electronics more and I have been trying to wave them off that and get them outside more…And now I’m kind of biting my tongue on it a little bit. We can’t go outside and do like we want to, and I don’t want to just have them sit in the house watching T.V. all day…But my little boy will say on that tablet all day, as long as it’s alive, he’ll put it on charge and eat with it and it just gets ridiculous. I really was trying to stray from that. I have a tendency to let them do would they want to now because I can’t give them anything else to do.

Jackson (28-years-old) recounted using this time with his son to prepare him for the biases he may experience as a young Black male navigating racism. He particularly wanted to ensure that his son knew to be aware of his Black maleness and how that is deemed as dangerous in certain situations:

I tell my boy all the time that you got to have it all together. They can slip like that, you see. But we can’t. We’re not going to get the same opportunities. We don’t have a chance to fuck up and be disrespectful and all that stuff. They’re not going to, they’re going to lock us up. Your first thing you do, bro, it’s not going to be no slap on the wrist. It don’t work like that out here. They will ship you off for anything, the first chance they get, they will lock your ass up. And then you’re a felon…He knows already… Because, I want him to understand…They’re so young. They’re his friends, but everybody’s not your friend, and you can’t go everywhere, and you can’t hang out with everybody and do what they do.

Adaptations such as these extended to the children’s education as fathers adapted to their children’s sudden need for homeschooling. Some fathers used this as an opportunity to spend more time with their children by helping them with their homework. Jefferson (29-years-old) recounted becoming more involved in his children’s education because of forced homeschooling:

With school being virtual we’re doing more at home activities with them instead of having them just sit around and do nothing. We are very strict about my children’s work and we are strict about their potential and their brain function. We are very into their schoolwork. We notify the teachers during this COVID… We try to interact with the school especially with their school teacher to make sure everything’s okay with school.

The sudden transition to homeschooling was difficult for many families as some fathers reported being ill-equipped to supplement their children’s education. As Bernard (26-year-old) recounted the difficulty of serving as an educational resource to his children:

I mean ain’t been in school in a minute, but it’s nothing too stressful that I can’t handle. I mean, brushing up on my math a little bit, it’s okay…If they did it by themselves honestly, no, I don’t think they would. If they got stuck on something there’s really nothing there to help them. They’d say to go back to the text and reread, but the kid has already read the chapter three times and he don’t understand it, he needs some help.

The impact of the pandemic on participants’ identity as a father was, it seems, a largely positive one. Men recounted having more time to get involved with their children recreationally and educationally. Participants also indicated that the pandemic allowed them to nourish their relationship with their children and further refine their identity as fathers.

#### Rona Romance

Intimate relationships were crucial in influencing participants’ experience of the pandemic. Several participants were cohabiting with partners or moved in with partners to stave off pandemic-related financial instability at the beginning of the pandemic. Two additional subthemes emerged: Relationship Enhancing Despite Adversity and Dating During COVID. For instance, Delroy (29-years-old) moved in with his girlfriend at the start of the pandemic to save money and support her:

Right at the pandemic. I’d made a decision that, um, I wanted to be here with her because, um, I didn’t wanna go back-and-forth from, you know, home to here, back. And she has a small child. So I made a executive decision to do that, and it has, it has worked. We worked through a lot of things… The pandemic has forced us to know each other—learn each other better.

This decision was a positive one, as Delroy (29-years-old) discussed, in our second interview, how his relationship had grown stronger throughout the pandemic:

Last month was a financial challenge, but we made it. But mostly, things have been good. Ups and downs, but we have really found a way to hope, hope and to disagree, agree to disagree, for the sake of peace…we communicate throughout the day, our schedules are hectic, so we make time…You know, like I said, I hate it had to be a pandemic to happen for us to be like that but we have had a more positive outcome than negative when it comes to, um, living together during this pandemic.

Several men in relationships also shared feelings as though their intimate relationship had improved due to the additional time they spent with their partners, such as Tyrone (28-years-old):

So me and my girlfriend, we live together, um, uh, I wouldn’t say it impacts it in a negative way, if anything, we’ve been able to talk to more and learn each other more because, for the past seven to eight months, we’ve been quarantined, like, just in, and, you know, she has a job, she ha- she works from home, so, if anything, we got to know each other a little bit more.

### Relationship Enhancing Despite Adversity

While men discussed the largely positive effects of the pandemic on their intimate relationships, relational challenges were also highly prevalent. For example, many couples had to alter their engagement in non-sexual intimacy (i.e., dating behavior) to be responsive to the restriction posed by the pandemic. Lemar (29-years-old) remarks how his inability to leave the house or take a break from one another has been associated with increased stress and subsequent fighting:

It’s been okay, you know, you get, you don’t really have that, that relief of being able to leave and just go somewhere, you know, it’s just like, you have to stay here and deal with it, and sometimes it works, sometimes it doesn’t…Sometimes she’ll think something’s wrong that she did, and it’s just me in my head, you know. And I’ll think she’s not supportive enough or something like that, and then we’ll just get into an argument and, sometimes it blows up, sometimes we’ll work it out, but you know, it’s just a lot on the mental…It’s a little of both [stress and pressure], honestly, ’cause we wouldn’t have the outlet if it were not for COVID, I think, to just get up and go, you know, enjoy each other outside of the house.

Despite this stress, several men recounted having to collaboratively work with their partners to alter the ways in which they managed their finances and spending behavior. For instance, Eric (27-years-old) stated:

I felt like we were spending a lot of money at the time on a lot of things that we really didn’t need. So one day, I decided to sit down with her and talk to her. And you know, we talked about the whole situation, about desires and all of that. And I told her, you know, a lot of people not working, a lot of jobs are being stopped at the time, you know? And I told her all the extra money that we’re spending and all the food that we buy; we eat out a lot, things like that, we had to stop doing, you know? Because we don’t know what’s going to happen next. You know, she told me she agreed. We sat down and thought about all the things that we were wasting money on, and we agreed not to waste money anymore. So that has helped us out a lot.

#### Dating During COVID

While few participants in this study were single, all who were single shared a common experience of finding it difficult to date during the pandemic. Leon (29-years-old) discussed how being a first responder during the pandemic limited his dating ability:

Dating has been, even as a first responder, it’s already hard to date a first responder because our s- schedules stay so wide array. Like, some days, we’ll work a 24, and we’re off for 12, and we’re on for another 24-or we’re on for another 48. And so you don’t, you know, you don’t get that with a partner, you don’t really, you know, they don’t, sometimes they don’t understand. It takes a special person to really understand. And so, even with that or, and then now, "Oh my God, are you around somebody that has COVID? Okay, um, yeah, we’ll just talk later," or, "Yeah, I’m not coming over." And you’re like, uh, there goes that one.

Intimate relationships, when present, had a positive effect on men’s lived experiences of the pandemic as partners collaboratively coped with the additional stress and burden of the pandemic; however, developing these kinds of relationships during the pandemic were often difficult.

### Essential Community

Men noted three significant sources of tertiary support: friends, God, and the church. Like men’s experiences with their family members, children, and romantic partners, interactions with these tertiary support systems were also heterogenic. These tertiary support systems contributed to sustaining men’s mental health despite the added adversity initiated by the pandemic. Three additional subthemes emerged: The Crew, Spirituality, and The Church.

#### The Crew

Friendships were often cited as the least affected interpersonal relationship in participants’ lives. As such, men recounted dedicating significant time to cultivating and perpetuating these relationships throughout the pandemic. For instance, several men were willing to make exceptions to their normative protective behaviors to socialize with their friends. During our first interview, when COVID-19 vaccines were not widespread, Delroy (29-years-old) recounts not wearing or requiring his friends to wear masks when they were hanging out:

I’m gonna be honest. When I’m with my close friends, we don’t, and I think it’s a trust thing. Because, um, I feel like it’s kinda like a break from the reality, honestly. Because we always have ’em on…Especially when we’re at work or we’re out into public. And I feel like that’s just a break a, a gasp of fresh air. And with my closest friends we don’t [wear masks] unless we’re out socially, but when we’re in a house together, we don’t. And it’s really just like a break.

#### Spirituality

In addition to their friendships, many men cited God as a significant source of support throughout the pandemic. Leon (29-years-old) articulates how his ability to pray and connect with God was critical to his ability to navigate the pandemic:

I learned that even when I think I’m at my lowest, even when I think it’s not possible that I could look within and keep pushing, keep praying, and keep pushing. I’m a guy that I got to give credit to God. If it wasn’t for God, I wouldn’t have made it through. There’s day that I really couldn’t even pray; I just cried. I was always taught tears are a silent prayer to God when you don’t know what exactly to pray. There’s praying days where all I can do is cry because I didn’t know what else to say, I didn’t know what else to do. Looking back on it, literally, it taught me to have faith more. It really strengthened my faith.

#### The Church

It is important to note that while men’s discussion of their relationship with God was common, they rarely associated themselves with a formalized doctrine or religion. Even when referencing the Church, men’s experiences of its function were more akin to a formalized social space than a respectful place of worship. For example, Leon (29-years-old) recounts how his inability to go to church and largely socialize with peers and extended family negatively affected his mental health:

I’m spiritual, I really used to enjoy going to church. I go to a church of about, probably about 1,100 people. A lot of my therapy was just going to church, seeing people at church, meeting people at church because I went to a church that is kind of world renowned known, um, and a lot of people come in to visit it. So, just meeting new people at church and just seeing different people and seeing church members used to be my therapy. And now, to going to church online and not seeing, you know, not experiencing having the feel of just being there and, and actually just participating and actually just going, so actually just being in the vicinity or being in the, in the midst of people, it’s so different, it’s like it’s almost depressing (laughs). So, not being able to actually get out and do stuff or see people or do things.

Men’s connection to tertiary sources of social support, such as friends, God, and religious communities, was integral to their ability to meet the demands of the pandemic. These support systems were so important that many men were willing to act outside of their normative protective behaviors to engage with these systems fully.

## Discussion

This qualitative study aimed to explore the effects of the COVID-19 pandemic on rural Black American men’s interpersonal relationships. Two waves of qualitative interviews were used to generate study data. Thematic analysis, informed by van Manen’s approach to hermeneutic phenomenology and principles of critical ethnography, was used to extract four essential themes from the data: (1) Familial Reorganization, (2) Adaptive Fatherhood, (3) Rona Romance, and (4) Essential Community. Findings suggest that the pandemic severely impacted participants’ interpersonal relationships with family members, children, and romantic partners. For some participants, the pandemic enabled them to improve these relationships; however, these enhancements were coupled with several notable challenges, including increased relational distress. The men overcame these challenges by changing their relationship with these interpersonal systems. Findings highlight the need for stakeholders to consider men’s relational health when developing and implementing pandemic recovery efforts, as it can significantly influence their ability to recuperate mentally and physically.

### Familial Reorganization

Familial relationships were a salient component of each participant’s lived experience, yet how these relationships changed largely depended upon the quality of the relationships before the pandemic. Some men articulated how the pandemic motivated them to reconnect with family members. These men’s experiences align with prior research indicating that some individuals went to great lengths to strengthen familial relationships because of the pandemic [63, 64]. Within the Black American community, it is common to utilize a mixture of Africultural and conventional coping strategies (e.g., problem- and emotion-focused coping) to address contextual stressors. Lewis-Coles and Constantine revealed that Black American men who perceived greater stress associated with community-level stressors were more likely to use collective coping strategies to manage the situation [65]. On the other hand, Black American women who perceived greater stress related to structural-level stressors were more likely to endorse collective coping strategies, cognitive/emotional debriefing, and spiritual-centered coping to deal with their difficulties [65]. As such, strengthening familial bonds and engaging collective coping strategies may be central in mitigating contextual stressors among Black American men.

Conversely, some participants reported feeling a growing distance within their familial relationships due, in part, to a lack of consistent in-person interfacing. While incongruent with much of the current research examining the impact of the pandemic on familial relationships, this finding is consistent with a broader literature documenting how contextual stress influences how family members interact with one another [66–68]. For instance, prior research suggests that adverse family circumstances such as financial stress and household chaos (e.g., a lack of routines) are associated with decreases in familial communication and connectedness [69, 70]. This is critical as family members may play a role in scaffolding each other’s pandemic experience. In other words, expending energy trying to reconnect or strengthen emotionally unsafe familial relationships may be more detrimental to an individual’s mental health than limiting their communication with certain family members. These data and our results suggest that participants may have viewed a small social network of positive relationships as more beneficial than having a large social network populated by neutral or even negative familial relationships. Given that many people are experiencing home confinement and potential isolation from other informal support systems (e.g., co-workers, friends), the conservation of positive familial relationships or the reduction of negative familial relationships are important processes for researchers and clinicians to explore in future research. Such data would particularly interest researchers and clinicians interested in leveraging social networks to disseminate information or interventions.

### Adaptive Fatherhood

In this study, fathering during COVID-19 presented a unique challenge and opportunity for men to engage with their children. On the one hand, fathers felt a sense of stress and anxiety about the health and wellbeing of their children, particularly related to how the pandemic may affect their social and educational futures. On the other hand, fathers leveraged their time at home to reassess and strengthen their relationships with their children. To our knowledge, only one study has examined the effects of the COVID-19 pandemic on men’s fathering. This study indicated that children’s emotional problems and inattention/hyperactivity levels significantly increased during the lockdown period [71]. The lockdown period was also associated with worsening parent-child communication and satisfaction [71]. Additional analyses highlighted that the principal predictor of paternal parenting stress was living in the regions most affected by the COVID-19 pandemic, followed by high levels of paternal anxiety symptomatology and high levels of worsening of the relationship with the child during the pandemic [71]. These data indicate the need for further research investigating the effects of the COVID-19 pandemic on fathers, as prior research has nearly exclusively focused on mothers’ experiences.

Future research examining the effects of the COVID-19 pandemic on Black American men’s fathering will require new conceptual frameworks that attend to the complexities intertwined with the intersectionality of Black American fatherhood. Current fatherhood theorization has largely overlooked the lived experiences of Black American fathers and has remained focused on White American middle-class men in monogamous marriages [72]. Consequently, contemporary understandings of Black American fatherhood are filtered through essentialist, Western, and middle-class theories that often result in stereotyping these fathers’ behaviors [73]. For example, the discursive stereotype of Black American fathers being “absent” and Black children being “fatherless” remains highly prevalent. In contrast to this discriminatory ideology, Levs notes that most Black American fathers (~ 2.5 million of around ~ 4.2 million) reside with their kids, even if they’re not married to their partner [74]. Furthermore, a recent Centers for Disease Control and Prevention (CDC) report indicates that Black American fathers, whether they live with their children or not, are more actively involved in their children’s lives than their counterparts of other races [75]. The report revealed that residential Black American fathers are more likely than fathers of other races to provide physical care (e. g. bathe, diaper, feed) for their young children, read to their children, and help their children with their homework—daily—than fathers of other races who also cohabitate with their kids [75].

The lack of critically informed conceptual frameworks concerning Black American fatherhood may also be related to the lack of research. For example, many articles cite the statistic that single women raise 70% of the Black American community; however, a critical examination of the source from which this statistic was derived revealed that when the original authors referred to *single women*, they were referring to *unmarried women* [76]. Being unmarried parents does not make a child automatically fatherless, as many unmarried couples live and raise children together, as demonstrated in this study. Furthermore, many studies of Black American fatherlessness mistakenly use residential status as their sole indicator [77]. This is problematic as fathers can remain highly involved in their children’s lives without having to live under the same roof. For instance, evidence suggests that non-residential Black American fathers are the least likely to report that they are not at all involved in the care of their children, including bathing, dressing, changing diapers, and playing with their children [75]. Black American fathers were also more likely to be involved in care, including reading to their children, helping them with homework, talking to them about their days, and taking them to activities, than Latinx or White American fathers who live apart from their kids [75]. The presence of Black American fathers within the home has profound implications for children’s development, particularly among young Black American boys. For instance, Black American fathers often engage in racial socializing practices that prepare their children for encounters with racism by adopting Black existentialist perspectives that emphasize self-determination and resilience as racially and politically motivated acts of resistance. As such, Black American fathers may play a very important role in communicating to their children the realities of racism while teaching cultural pride and providing strategies for overcoming racial barriers that are qualitatively different from Black American mothers’. However, research in this area remains scarce.

The combination of a lack of critically informed conceptual frameworks and empirical research has resulted in the obfuscation regarding how structural racism and inequality influence Black American fathering. Emerging research indicates that structural racism may disincentive socioeconomically struggling parents from living together or getting married as it would limit or eliminate their access to key social safety nets, such as Supplemental Nutrition Assistance Program (SNAP) or public or subsidized housing [78]. As discussed previously, Black Americans are more likely to live in racially segregated communities where access to social safety net programs may be crucial for children’s survival [79]. Thus, many Black American parents must weigh the pros of being married and living together against the cons of not having reliable access to resources. By stepping outside the pervasive White heteronomative, monogamous framework of fatherhood, researchers may gain a more nuanced and accurate understanding of Black American men’s fathering.

The future examination of the effects of the COVID-19 pandemic on Black American men’s fathering and the father-child relationship should be of distinct public interest as research indicates that young Black American children have been uniquely affected by the pandemic. For instance, recent evidence indicates that Black and Latinx American households were less likely to have reliable access to electronic devices that would best facilitate digital learning, thus reducing the number of hours children spent in the remote classroom compared to White American households [80]. The COVID-19 pandemic’s longer-lasting legacy could be, in part, the harmful consequences for children who do not fully recover the core skills and abilities they were developing before the pandemic. Students with learning, as well as developmental disabilities, could be particularly vulnerable, given that the intensity of these services—including inputs such as speech therapy and smaller in-person teacher-student interactions—are nearly impossible to recreate through virtual platforms amid the pandemic [81]. Considering the immense potential effects of the pandemic on Black American children’s downstream health and wellbeing, further research might explore how fatherhood involvement, or the quality of the father-child relationship may mitigate the determinantal effects of the pandemic.

### Rona Romance

Participants’ experiences of trying to maintain a romantic relationship during the COVID-19 pandemic were largely positive as they recounted collaboratively coping with their partners to address the additional stress and burden of the pandemic. These findings contribute to emerging research documenting the paradoxical effects of the COVID-19 pandemic on couples’ functioning and wellbeing [82]. One line of research indicates that the pandemic has harmed couples’ functioning and wellbeing. For example, Merolla et al.’s assessment of how the COVID-19 pandemic altered the day-to-day relational experiences of 112 adults indicated that the quality of romantic relationships was negatively impacted by the fear of contracting the COVID-19 virus, depression, worry, and rumination about the pandemic [68]. Participants in this study also reported more isolation and a lower ability to reduce conflicts [68]. Another, more developing line of research indicates that the pandemic may have positively affected some couples. Donato et al. (2021) demonstrated that COVID-19 pandemic-related concerns led to more explicit stress communication related to higher perceived partner dyadic coping [83]. These data indicate that while pandemic-related concerns were negatively linked to psychological wellbeing, they also improved dyadic coping [84]. Future research should examine these processes among Black American couples as they (a) are highly under-represented in relational research and (b) may evidence unique cultural methods for collaboratively coping with stress and anxiety.

### Essential Community

Participants described how tertiary sources of support (e.g., friends, God, and the Church) were essential to maintaining their mental health. The finding of participants’ using friendships as sources of support is following robust prior research illustrating the safety and efficacy of peer support in enhancing an individual’s experiences of hope, quality of life, self-esteem, and social functioning in the face of the significant context stress, such as the COVID-19 pandemic [85–87]. Participants further recounted that spirituality, in their relationship to God and the Church, was described as a salient social determinant of health and sources of support. However, men drew clear distinctions between God and the Church. Participants more frequently discussed turning to God for support and using the Church as a space for building a sustainable community and social interaction. These findings contribute to ongoing research regarding the complex spiritual, communal, and political roles of the Black Church in Black Americans’ experiences of the COVID-19 pandemic. Prior research indicates a general decline in Americans’ endorsement of and engagement with organized religion over the last 20 years [88, 89]. However, another line of research suggests that Black Americans, more than any other racial/ethnic group, perceive religion as an important element of their daily lives [90, 91]. This disparity might be related to misunderstandings regarding the role of the Black Church and personal spirituality in the lives of Black Americans.

Dating back to the Antebellum South, the Black Church has been a central pillar in Black American history, identity, and social justice [92]. To be clear, there is no single Black religion. Still, the traditions and faiths that fall under the umbrella of Black American religion, particularly Christianity, constitute two stories: one of a people defining themselves in the presence of a higher power and the other of their journey for freedom and equality in a land where humanity is often denied to them. Collectively, these churches makeup among the oldest institutions created and controlled by Black Americans and, as such, are more than just simply places of worship [93].

Although Christianity had been practiced in many parts of Africa for centuries, many enslaved Africans were forcibly converted to white slaveholders’ conceptualizations of Christianity to quell chances of future rebellion. As such, enslaved Black Americans synthesized their indigenous religious expressions with the theology thrust upon them, creating a unique cultural expression of Christianity [94]. Consequently, Black American churches became centers for spiritual, communal, and pollical organization and unity [95]. After the Civil War and the emancipation of Black Americans, the shared faith between Black and White American Christians could have presented an opportunity to integrate these different religious expressions [94]. However, institutional racism within the church and broader society first created invisible institutions and later parallel religious institutions with separate historic Black denominations like the African Methodist Episcopal Church, National Baptist Convention, and Church of God in Christ as well as within predominately White denominations like the United Church of Christ, United Methodist and Southern Baptist [95].

Despite institutional racism, the Black Church has influenced nearly every chapter of the Black American story, both for believers and nonbelievers. It will most likely continue to serve several key functions, including a spiritual center, a communal meeting place, and organizing space for political activism [93]. The multifaceted role of the Black Church was exemplified early in the pandemic when they often took a more somber view of its effects and voiced concerns for their attendees’ public health and civic responsibility [96]. Meanwhile, many White clergies voiced concerns for constitutional liberties and freedom to worship as an important first amendment right and the need to continue the regular in-person pattern of worship [97]. Gates Jr posited that Black American and conservative White American church leaders may have weighed the scientific consensus and their religious mandate to congregate differently, with the latter dismissing scientific knowledge that could not be reconciled with their religious belief [95]. Gates Jr further argued that because of the Black Church’s multifaceted role, Black American clergy members forefront of their community’s material grief, loss, and suffering over any personal or religious attitudes towards the pandemic [95]. I would like to note that this trend towards promoting communal health over individual ideologies mirrors that of participants in this study, as many were vaccinated against COVID-19 despite their hesitancies. To develop a fuller picture regarding the effects of the contextual crisis on Black American men’s health and wellbeing, additional studies will be needed to delineate to what degree—or under what circumstances–the Black Church vs. personal spirituality protects men against acute stressors.

### Strengths & limitations

Several strengths and limitations of the present study should be noted. A key strength is a focus on the lived experiences of rural Black Americans, as they are often neglected in empirical research concerning both Black Americans and rural health. Focusing on this highly vulnerable community provided novel insights into rural Black American men’s health and wellbeing that can be leveraged to develop multisystemic pandemic recovery interventions. This strength also serves as a limitation as the focus on Black American men living in rural communities in the South may not be transferable to men living in urban settings or other regions of the United States. Another limitation of this study was that while all men were invited to participate in the second wave of interviews, only eight chose to participate a year later. This limited our ability to conduct member checks with all participants systemically; however, there was a minimal degree of divergence in the second wave of interviews, suggesting that additional interviews would not necessarily elicit new information. We did not ask men to disclose their sexual orientation, limiting our ability to investigate or identify differences in lived experiences based on participants’ sexual orientation. Last, due to the establishment of a prolonged participant-research relationship, it may be possible that there was a responder or social desirability bias as participants may have sought to provide answers that they felt we would be the most amenable to. However, this prolonged participant-research relationship may have also made participants feel more comfortable speaking candidly and honestly. We took active steps to avoid such biases by consistently offering participants unconditional positive regard throughout our interactions while avoiding leading questions and allowing participants to speak until the retelling of their experience was completed. These limitations notwithstanding, the present study had several strengths that provided novel insights into how the COVID-19 pandemic has influenced rural Black American men’s interpersonal relationships.

## Conclusion

The current study examined how the COVID-19 pandemic influenced rural Black American men’s interpersonal relationships. Study findings revealed that while the COVID-19 pandemic evidenced salient positive effects, such as developing stronger interpersonal relationships, these positives came at the expense of increased relational conflict and intrapersonal stress. These effects varied by the type of relationship being scrutinized. For instance, peer relationships experienced fewer challenges and changes than familial relationships. Findings indicate that the effects of the COVID-19 pandemic, even after its conclusion, will continue to influence rural Black American men’s relational health as participants develop new patterns of interpersonal interaction to respond to the demands of the pandemic. As such, rural Black American men’s differential experiences of the pandemic must be considered by scholars, clinicians, or interventionists interested in developing efficacious pandemic-recovery interventions and policies. Future research is needed to monitor and explore the downstream effects of the COVID-19 pandemic on rural Black American men’s health and wellbeing.

## Supporting information

S1 Table In-depth participant profiles. (DOCX)

S1 File Wave 1: Interview protocol. (DOCX)

S2 File Consolidated criteria for reporting qualitative research checklist. (PDF)
