# Supplementary material for: Systemic effects of the COVID pandemic on rural black American men’s interpersonal relationships: A phenomenological examination
Source: PLoS One. 2024 Apr 17;19(4):e0297876. doi: 10.1371/journal.pone.0297876 (PMC11023195; doi:10.1371/journal.pone.0297876)
Supplement: S1 Table — (DOCX) [file pone.0297876.s001.docx]

| Table 2. Participant Profiles | |
| --- | --- |
| **Participant** | **Vignette** |
| **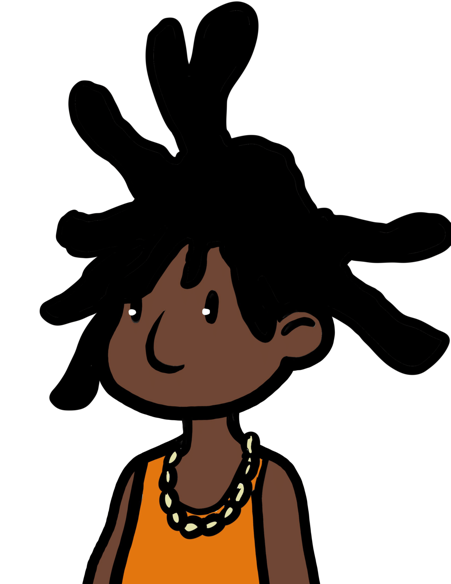**  **Bernard** | Bernard was a 26-years-old father working in landscaping. He reported feeling as though his life had not changed much due to the COVID pandemic but felt a bit of stress regarding not being able to see family members who were struggling with a diagnosis of COVID. He discussed not feeling afraid of contracting the virus himself because his job did not require him to come in much contact with other people. For Bernard, the COVID pandemic was the most impactful on his children’s lives as they were not allowed to attend school or socialize with children their own their ages. He recounted feeling concerned about the long-term effects of the social isolation on his children’s wellbeing and development. |
| **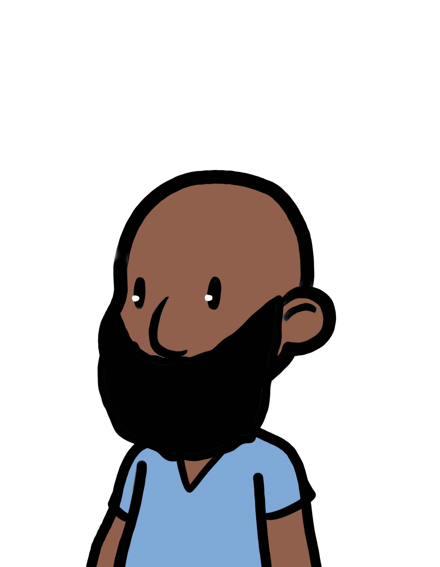**  **Ben*** | I met Ben when he was 27-years-old and working in the insurance industry. He was the fourth of five children and was currently single. According to Ben, he was not raised in a very affectionate family and moved away shortly after graduating high school. While he lacks strong connections with blood relatives, he has developed a strong found family with friends. During our second interview, he discussed the ongoing challenges of working from home, anxiety about prospect of returning to the office, and visiting friends in Europe when lockdown regulations eased. |
| **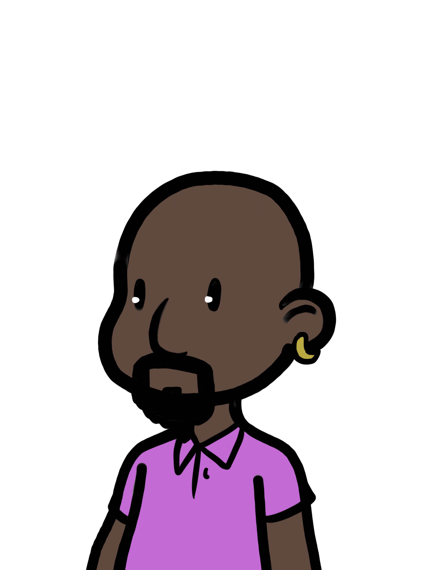**  **Delroy*** | Delroy was a 29-years-old college student pursuing his bachelor’s degree at the time of our first interview. He was largely raised by his grandparents and is the eldest of six siblings. At the start of the pandemic, he was laid off from his job and struggled with distance education but eventually learned to adapt to a new way of learning. Early in the pandemic he moved in with his girlfriend to help her with childcare as she was required to work outside the home. While he felt daunted about assuming the role of a stepfather, he was eager to serve in a support role. In our second interview, Delroy recounted being focused on completing his bachelor’s degree and achieving financial stability despite issues-related to the COVID pandemic limiting his employment prospects. |
| **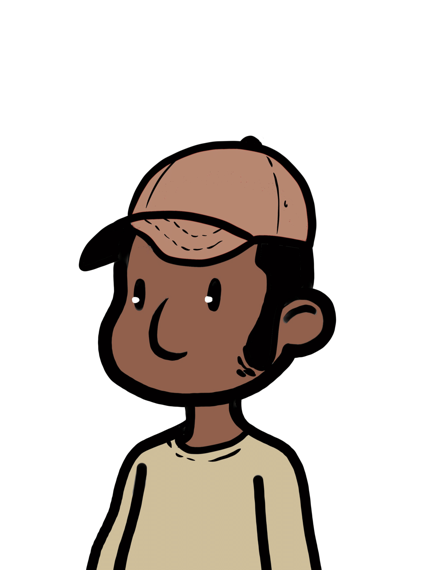**  **Elias** | 27-years-old Elias described himself as an intellectual, father of two who enjoyed reading philosophy, gardening, and being in nature at. At the time of our interview, he had recently proposed to the mother of his children. He worked in the construction industry. He unfortunately was unable to finish college due to the birth of his first child which required his early entry into the workforce, but he was interested in returning to school to complete his degree. |
| **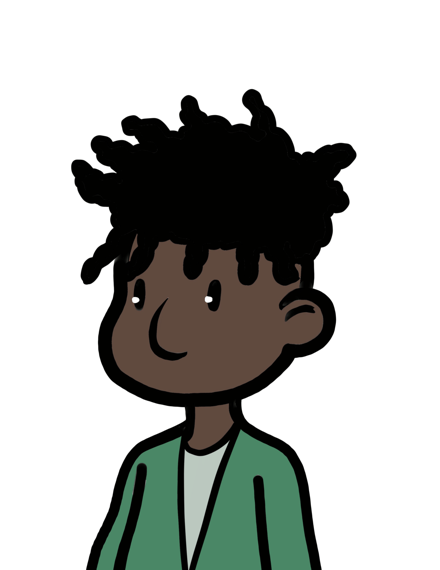**  **Eric** | Eric was a 27-years-old working in the automotive industry. He was the eldest of 13 siblings and felt pressure from both sides of his family to be a good-role model. Due to his parents separating at an early age, he was largely raised by his grandparents, who were his primary caregivers, and was extremely close to them. At the start of the pandemic many family members lost their jobs (e. g., his mother, grandparents, sisters) and he felt a responsibility to financially contribute to their stability, even though he was making less money on average. |
| **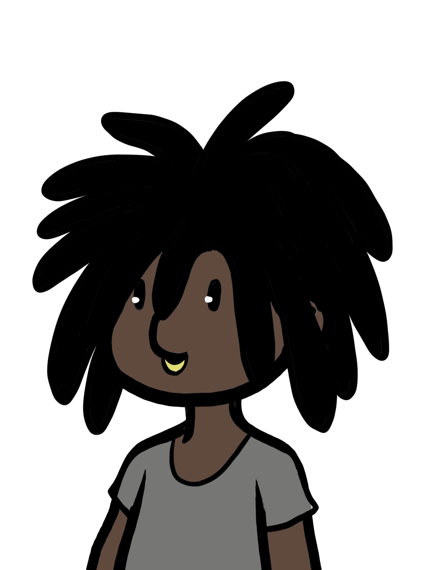**  **Jackson*** | Jackson was a 28-years-old father of two. He had a close relationship with his parents and had one sister. Recently, he started a clothing business with a friend that sold clothes, hats, and beanies, but primary worked in the logistics industry. While he experienced a downturn in income at his primary job, his income was supplemented by an uptick in sales in his business. During our second interview, Jackson spoke at length about his distrust of the COVID vaccine and feared the undiscovered downstream effects of the vaccine. |
| **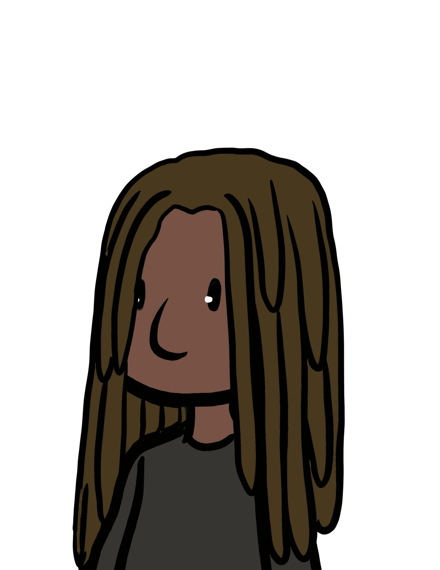**  **Jakeem** | Jakeem was a 28-years-old who had recently moved to a larger city in his area. When we met, he had recently started working overnight at a retail store taking inventory and stocking shelves. He had lost his previous job due to the pandemic forcibly closing his last place of employment. Jakeem hobbies included gardening and creating different types of music including R&B, hip hop, rock, alternative rock, and jazz. He reported spending the bulk of his time outside of working helping his only brother create music. |
| **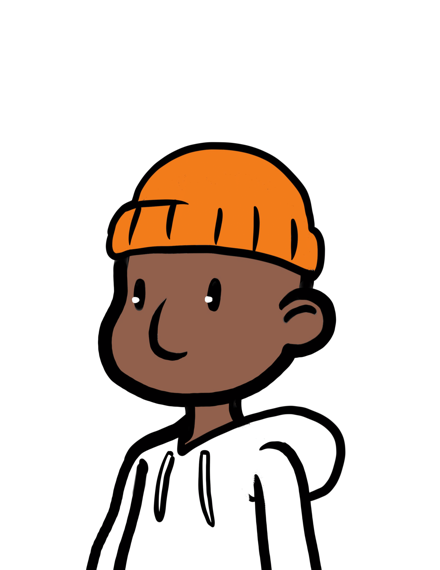**  **James** | James was a 27-years-old father of 7 who worked in the automotive industry. He was the youngest of 4 children and was extremely close to his mother and father, even though they were no longer together. He was the brother of another participant, Jefferson. Their interviews were not connected in anyway and took place at different time. James described himself as a dedicated father and sought a job that would allow him to see all his children since two of them are spread across neighboring states. On his trips he would often stay with the mother of his children so he can spend time with his children. Throughout our interview James expressed deep concern for the safety of his children and took considerable precautions to decrease his likelihood of getting and spreading COVID-19. |
| **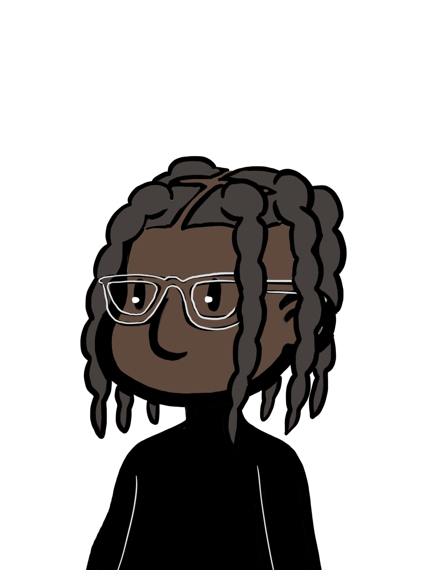**  **Jefferson** | Jefferson was a 29-years-old father of 3 sharing a home with the mother of his children whom he had been with for 11 years. He described himself as a positive person who was dedicated to ensuring that his children had opportunities that were denied to him. He was the second of 4 children, with his eldest brother being another research participant, Jefferson. Their interviews were not connected in anyway and took place at different time. Towards the start of the pandemic, he, and his girlfriend both lost their jobs and were forced to move in with his family, whom he is close to. At the time of our interview, they had just moved into their new place a few and were in the process of rebuilding their lives. |
| **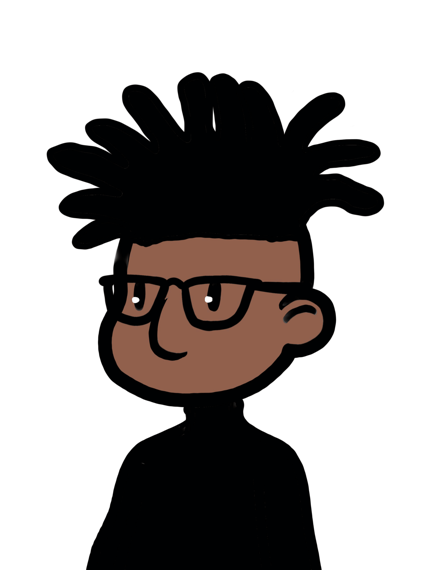**  **Lemar*** | When I first met Lemar, he was a 29-years-old single man working in the criminal justice field. At the time of our first interview, he was visiting his parents for an extended stay to support his mother through her battle with a debilitating illness. She unfortunately passed away shortly before I sat down with him again for our second interview. Lemar reported to be a very active participant in the rearing of his only younger brother, as their father had slowly grown emotionally distant from the family as his mother’s health to decline. He also had a long-term girlfriend. Their relationship ended between interviews due to issues related to the pandemic and his own growth and development. During our second interview, Lemar was focused on rebuilding a normative sense of self and finding balance following the death of his mother. |
| **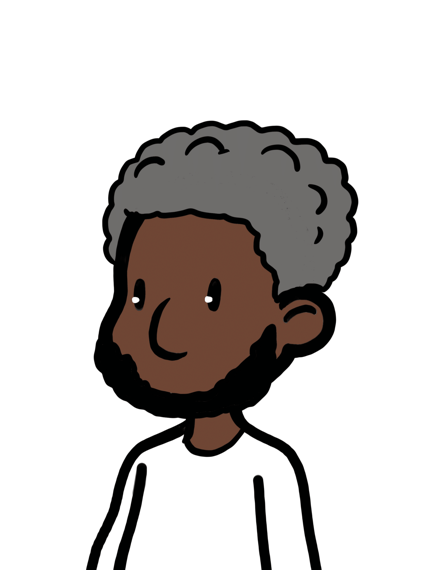**  **Leon*** | Leon was a 29-years-old college graduate currently working in the public health industry. He was primarily raised by his grandmother since, his parents were teenagers when they gave birth to him. The recent passing of his grandmother a few years ago created distance within the family system due to her being the central promotor of their connections. During our first interview, he recounted his harrowing experience with suffering through COVID to the point of being intubated. He recovered but still experienced some of the lingering effects. By the time of our second interview, he had undergone a drastic job and life change as he re-evaluated his quality of life and made changes to align himself with his preferred way of being. |
| **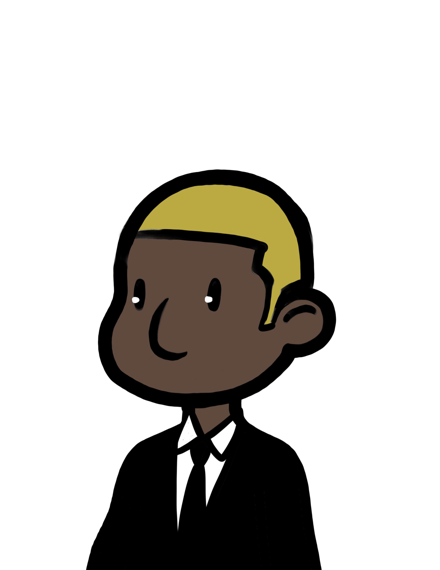**  **Luther** | I met with Luther when he was 28-years-old and working in the healthcare industry. He was one of seven children and was extremely close to his mother. As a teenager and young adult, they butted heads a lot, but they grew to respect each as adults and have a closer relationship than ever before. While Luther acknowledged the additional stress the pandemic elicited, he also noted the peace the pandemic provided him by allowed him to slow down his life by limiting his daily activity. |
| **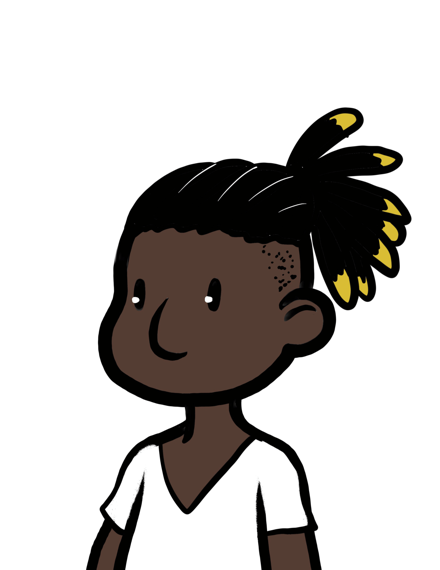**  **Markus** | Markus was a 26-years-old father of one. He worked in retail and was currently in a romantic relationship with the mother of his child. He lived with his mother who he noted to be a very good support system for him and his child. As a result of him living in a more rural area, much of his daily life did not change because of the pandemic but he did recount procedures at work changing near constantly, due to his place of employment being open throughout the pandemic, and the stress of ensuring that he was always in compliance. |
| **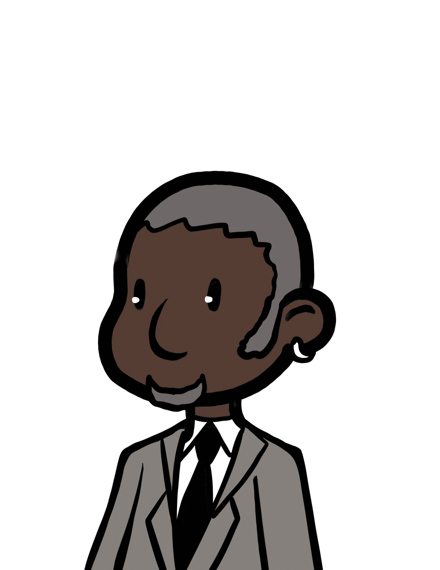**  **Samuel** | Samuel was a 28-years-old father of one. He was currently unemployed and searching for work but found it difficult to secure stable employment due to the pandemic. He recounted having submitted numerous applications with no callbacks or interviews. During our interview Samuel expressed concern regarding the validity of the virus as being deadly but took precautions, via social distancing and wearing masks, to ensure the safety of his child. |
| **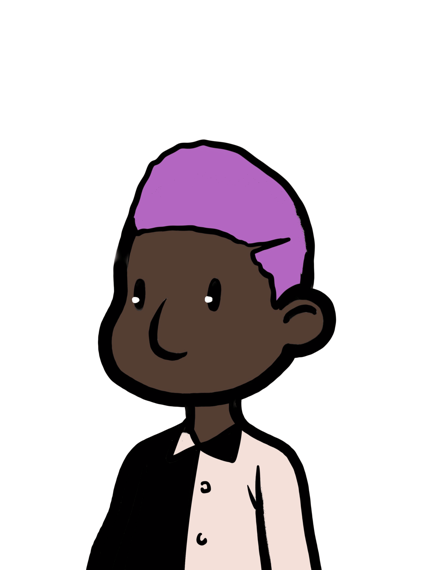**  **Tyrone*** | Tyrone was a 28-years-old high school sports trainer. He had one younger brother, with whom he connected with over sports. In our first interview, he recounted currently purchasing a home with his long-term girlfriend after long periods of renting. Unlike other industries, he recounted significant increases in revenue and clients as families focused their energy on developing their children’s skills while being forced to do distance-learning. During our second interview, he expounded upon how his industry was affected by the pandemic and how some promising players were suffering from a reduction in recruitment resources, which many of whom were relying upon to cover the costs of higher education. |
| **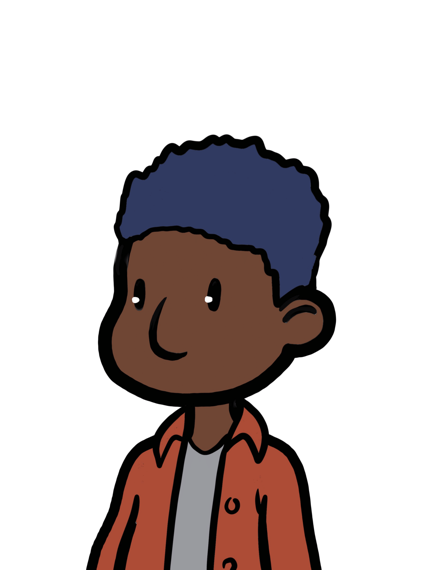**  **Victor*** | Victor was a 27-years-old mental healthcare worker. He recounted having a passion for working with children in the juvenile detention system. During our first interview, he had recently moved back in with his mother and father with the intention of purchasing a house shortly afterward. However, the pandemic regrettably derailed these plans by raising local housing prices and reducing his annual income. By the time of our second interview, he had moved out of his parents’ home and into his own apartment. Victor reported having a mild case of COVID towards the beginning of the pandemic and contracted it again before our second interview. Throughout both of our interviews, Victor was highly concerned about COVID because he worked closely with children, who struggled to follow protective protocols, and had lost friends to the virus. During our second interview, Victor recounted not having received the hazard pay that he was promised at the start of the pandemic, despite other departments in the same organization having received their pay. |
| **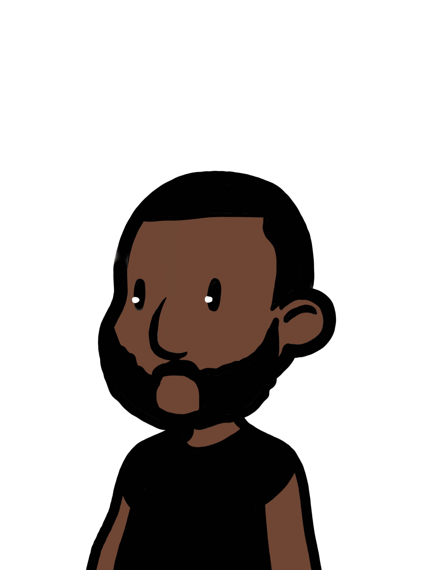**  **Virgil*** | I first met Virgil when he was 29-years-old educator. Virgil was the youngest of 5 children and recounted having a very closely knit together family. As a teacher, Virgil discussed the difficulties of supporting children and parents through the distance learning transition but highlighted the importance of being flexible in his approach to teaching as he acquired COVID in-between our first and second interviews and was required to work from home. Across interviews, Virgil expressed his concern for his student’s academic futures as he found it difficult to virtually track their progress and development. This concern was particularly pronounced in our second interview as he was in the midst of recovery from COVID and was preparing to return to work after being on medical leave for a month. |
