# Supplementary material for: Systemic effects of the COVID pandemic on rural black American men’s interpersonal relationships: A phenomenological examination
Source: PLoS One. 2024 Apr 17;19(4):e0297876. doi: 10.1371/journal.pone.0297876 (PMC11023195; doi:10.1371/journal.pone.0297876)
Supplement: S1 File — (DOCX) [file pone.0297876.s002.docx]

## Supporting Information File 1

Wave 1 Interview Protocol

***Before recording has begun:***

***Script***

Welcome and thank you for your participation today. My name is Michael Curtis, and I am a researcher at the University of Georgia, and I will be conducting your interview today. At this time, I am not recording and would like to take the time to answer any questions you may have before we start the interview. [Pause for answering questions]

If there aren’t any further questions, I am going to start the recording now.

***Introduction – talking points:***

- COVID 19 pandemic is having a tremendous impact on people’s lives across the world
- Black Americans are disproportionality susceptible to exposure
- We believe that what young Black men are struggling with is important and we want to hear about and understand how the pandemic is affecting Black men’s lives
- Personal connection to the research (creating space for participant to vent to us about their experience)

***Script***

The interview should last between 60-90 minutes. There are no ‘right’ or ‘wrong’ answers to these questions and if at any time during the interview you wish to stop the recording or the interview, please feel free to let me know. I will start the recording now.

***[Start Recording]***

***Conversation area I. Social Location. Who are our participants and what are some of the strengths and challenges of their contexts?***

Subtopics to cover

- Who is the participant?
- Describe the participants social ecology
- Who is important to the participant and why?

Potential Questions to Advance Conversation

- Tell me a little bit about yourself and your family.
- What it’s like living in your neighborhood?
- Who are you closest to?
- What do you do for a living?

***Conversation area II. COVID -19 and general health concerns. What are the participants’ (a) understanding of what the virus is and its consequences, and (b) major health concerns related to the virus?***

Subtopics to cover

- Experience with the virus
- COVID knowledge and sources of knowledge
- Perceived susceptibility
- Precautionary behavior

Potential Questions to Advance Conversation

- Tell me what you’ve heard about COVID.
- Do you know anyone who’s gotten COVID?
- Where did you/are you getting your information about COVID?
- How worried are you that you or someone you know will get it? Why do you worry more or less about specific people?
- What are you doing to be healthy?
- Would you be able to go to the doctor/get tested if you were to get sick?

***Conversation area III. How has the pandemic influenced the participant’s work life? What real- world changes have the participant experienced in regard to work?***

Subtopics to cover

- Changes in work life, comfort in the workplace, employment challenges
- Changes in access to resources (i. e. food, health care, transportation)
- How has COVID-19 impacted you, your family's, or your friends' access to resources such as food, health care, and transportation?

Potential Questions to Advance Conversation

- How has work changed for you? How have you been able to make ends meet?
- Did you receive the stimulus check? Was it helpful?
- What kind of things is your employer doing to support you during the pandemic?
- Do you feel comfortable working outside your home?
- What changes have employment or income caused to your household?

***Conversation area IV. Interpersonal relationships. In what ways has the pandemic brought out positive and negative aspects of their relationship with their significant others, children, family, and friends?***

Subtopics to cover

- Changes in household composition and behavior
- Changes in relationships with family members and friends
- Changes in leisure time, how it is spent

Potential Questions to Advance Conversation

- What has it been like trying to parent during COVID? What do you do with your children when you have to leave the house?
- How often are you able to see your family and friends? What are some of the things you talk about when you are together?
- Due to the virus, what sorts of things have you and your friends and family begun or stopped doing?
- How would you describe what is happening to your relationships with your friends and family as a result of COVID?
- When you think about your family and friends going through COVID, what are some of the experiences you have had?
- How have your relationships changed with romantic partners/children/parents/friends?

***Conversation area V. How is the participant coping/dealing with the changes to his life? In what ways has the pandemic brought out positive and negative behaviors and feelings?***

Subtopics to cover

- How stressful is it and how is the participant dealing with the stress?
- Negative coping, ways that stress is getting the best of people
- Positive coping, ways that participants “fight back” to maintain health and well being
- How has the participant’s life prepared him well or poorly to deal with the stress/changes of the pandemic?

Potential Questions to Advance Conversation

- What do you do to relax or have fun? Has this changed due to COVID?
- What’s it like having to wear a mask when you go out in public?
- How do you feel people are reacting to having to wear a mask?
- Who is and isn’t following the COVID recommendations?
- Did you live in a town that was under curfew at one time? What was that like? Did you have any interactions with police during that time?
- Walk me through a normal day/week for you. What do you find yourself doing more or less?
- Many people are feeling added stress and pressure due to COVID. Are you feeling any of that extra stress or pressure? Why or why not.
- Are you or the people around you worrying about things more than usual?
- How has it been not being about to get a haircut?

***Conversation area VI. Participant as consultant on the pandemic. This set of questions ask the participant to provide their expertise on the course of the pandemic, coping with the pandemic, and its effect on the Black community***

Subtopics to cover

- The consequences of the pandemic on people’s lives long-term
- What has been learned and advice for others in dealing with the pandemic
- The effects on the Black community

Potential Questions to Advance Conversation

- When all is said and done, how might COVID effect the world moving forward?
- The news is saying that Black people are more likely than White people to get COVID. Do you agree? Why or why not.
- How might COVID be affecting Black men differently than other men or Black women?

***Script:***

That concludes our interview. Thank you for taking the time to share your experiences with me. I look forward to our second interview in about 6 months. I will end the recording.

***Once the recorder has been turned off:***

***Script:***

Again, that you for participating. Before we end, I’d like to know if you have any final questions about the interview or the study itself?

*Verify the address for the incentive check.*
