# Supplementary material for: Systemic effects of the COVID pandemic on rural black American men’s interpersonal relationships: A phenomenological examination
Source: PLoS One. 2024 Apr 17;19(4):e0297876. doi: 10.1371/journal.pone.0297876 (PMC11023195; doi:10.1371/journal.pone.0297876)
Supplement: Attachment — Submitted filename: Review Results Dec2023.docx [file pone.0297876.s004.docx]

**Review Results**

**Article title:** Systemic effects of the COVID-19 pandemic on Rural Black American Men’s Interpersonal Relationships: A phenomenological Examination

Manuscript Number: PONE-D-23-31672

| **Review Comment** | **Recommendations** |
| --- | --- |
| Misarrangement of Key words | Please ensure Key words are arranged in an alphabetical order. |
| At line three (3) of the abstract, U.S should not be abbreviated  Sub-themes numbering | Please provide full meaning to U.S instead of the abbreviation in the Abstract  Also remove the numbers attached to the sub-themes at the abstract |
| At the abstract section, line 5, this study aimed to “qualitatively explore the effects” | Please remove the “qualitatively” and maintain the “explore” |
| No recommendations provided at the abstract | Please state at least two recommendations (be specific) in the abstract section rather than presenting it as “discussed” |
| Participants biodata missing | Authors should provide a presentation of biodata of study participants at the finding’s column. |
| At the findings section, direct quotations were not supported with participants details, | Authors to take critical look at the direct quotations at the findings section and support quotes appropriately with participants details.  This runs throughout the findings section and authors should pay critical attention to it.. |

Impression: Very insightful and educative study, however authors need to critically address concerns raised at the entire finding sections in addition to the minor comments raised in the study.
