# Supplementary material for: Systemic effects of the COVID pandemic on rural black American men’s interpersonal relationships: A phenomenological examination
Source: PLoS One. 2024 Apr 17;19(4):e0297876. doi: 10.1371/journal.pone.0297876 (PMC11023195; doi:10.1371/journal.pone.0297876)
Supplement: Attachment — Submitted filename: Revision Letter 1.docx [file pone.0297876.s005.docx]

Dear Dr. Editors & Reviewers,

Thank you for the excellent news that we have been invited to resubmit our manuscript, “*Systemic Effects of the COVID-19 Pandemic on Rural Black American Men’s Interpersonal Relationships: A Phenomenological Examination*” for exclusive consideration in the *PLOS One*. Below, we detail our revisions and responses, in bold, to each concern the reviewers raised.

**Reviewer 1**

**Comment:** The chosen theoretical frameworks were well justified to inform your research approach. Data analysis was described adequately. It is unclear the study timeline with the description of "The COVID-19 pandemic occurred after Wave 4 of data collection was completed." Does this mean your study occurred before COVID-19? The explanations for the different waves were confusing. It appears to me that only waves 1 and 2 were included in this study, and that AMP includes all 6 waves. Please provide some further explanation of the waves of data collection in your methods section and what the relevance of the different waves are to this particular study. What were the purposes of each wave, and why did interviews occur during various waves of your study?

**Response:** We have revised the Participants section to add additional context concerning the larger quantitative study within which the current quantitative study took place. We also clarified the timeline concerning when waves 1 and 2 of interviews took place. This section now reads:

*The COVID-19 pandemic occurred after Wave 4 of quantitative data collection was completed. The first wave of interviews was conducted from April 2020 to December 2020, while the second wave was conducted from September 2021 to October 2021.*

**Comment:** Was Table 1: demographic information collected during interview or on a survey form? This is not described. Assuming this is done on a survey format since there was missing data in the response.

**Response:** That is correct, demographic information was calculated using survey data from Wave 4 of quantitative data collection. We have clarified this language on page 11. It now reads as:

*The COVID-19 pandemic occurred after Wave 4 of quantitative data collection was completed (i.e. January 2020). The first wave of interviews was conducted from April 2020 to December 2020, while the second wave was conducted from September 2021 to October 2021.*

**Comment:** There are some abbreviations not abbreviated correctly (e.g. CDC, page 31; U.S. was abbreviated then spelt full form in Strengths & limitations, page 37). Typos in the in text citation (e.g. Robins et al., 2023, page 9). Contractions noted (e.g. "they're", pages 31 & 32). Some errors in the reference list (e.g. Allen, Q 2016, journal name and issue were not italic. Some links to references were not provided (e.g. Hardy & Logan 2021 <https://www.brookings.edu/articles/the-way-back-assessing-economic-recovery-among-black-americans-during-covid-19/>).

**Response:** We have thoroughly reviewed the manuscript and references section to correct these errors and have corrected any found mistakes.

**Reviewer 2**

**Comment:** In the background, the author provided sufficient information to establish the research gap fully. However, aside from the theoretical frameworks presented, it would have been better if the author included the philosophical underpinnings, which are particularly important in qualitative research because they influence how researchers approach their research questions, collect and analyze data, and interpret their findings.

**Response:** We have added a new section entitled “Paradigmatic Orientation” that articulates that critical post-modernism and social constructionism served as guiding philosophical paradigms that guided the development, and implementation of the current study (see pages 6-8).

**Comment:** In the methods section, the author wisely used a qualitative approach in this study as it allowed for a rich and nuanced understanding of the experiences of Black American men. Moreover, information about the participants and data collection and analysis were included. However, I am concerned with using a hermeneutic phenomenological qualitative approach since there is a potential for subjectivity. In hermeneutic phenomenology, the researcher is an active participant in the research process. Hence, the researcher’s own experiences and background might have influenced his interpretation of the data (e.g., Queer, Black American man who grew up in a low-resource environment; has made me keenly sensitive to the systems of oppression; I was, and remain, deeply connected to the men’s lived experiences highlighted in this study). While the author mentioned under the subheading trustworthiness about having a reflexive attitude and maintaining a keen awareness of the researcher’s biases and preconceived beliefs, it is unclear how he specifically did this throughout the data-gathering process. It is unclear how reflexivity and bracketing were done.

**Response:** Thank you for your insightful comment. While bracketing is a central component to many qualitative approaches it was purposely not integrated in this study or analysis. As articulated by Madison and the tenets of critical ethnography, the notion that a researcher can separate their lived experiences from that of their participants is unreasonable at it would (a) ignore my (MGC) status as an insider within the Black American and male-presenting communities and (b). Instead, I leveraged my status to create rapport with participants and cultivate and maintain a sensitivity to the power positions, choices, and effects that my lived experiences had on the research process. This sensitivity was documented via consistent memo writing throughout the data collection and analysis process. Externals audits by secondary researchers were utilized to ensure that these dynamics did not negatively influence the research process. We outline this process on page 18.

**Reviewer 3**

**Comment:** 2. At line three (3) of the abstract, U.S should not be abbreviated Sub-themes numbering: Please provide full meaning to U.S instead of the abbreviation in the Abstract. Also remove the numbers attached to the sub-themes at the abstract

**Response:** This has been corrected.

**Comment:** 3. At the abstract section, line 5, this study aimed to “qualitatively explore the effects”: Please remove the “qualitatively” and maintain the “explore”

**Response:** This has been corrected.

**Comment:** 4. No recommendations provided at the abstract: Please state at least two recommendations (be specific) in the abstract section rather than presenting it as “discussed”

**Response:** We added two recommendations. The final sentence in the abstract now reads:

*Future research should be dedicated to (1) investigating the effects of the COVID-19 pandemic on fathers, as prior research has nearly exclusively focused on mothers' experiences and (2) delineating protective effects of rural Black American men’s involvement in the Black Church from their individual spiritualities to gain a more comprehensive understanding of the influence of contextual crisis on their long-term health and wellbeing.*

**Comment:** 5. Participants biodata missing: Authors should provide a presentation of biodata of study participants at the finding’s column.

**Response:** No biological data was collected was collected in this study so there was no biological data to report.

**Comment:** 6. At the findings section, direct quotations were not supported with participants details: Authors to take critical look at the direct quotations at the findings section and support quotes appropriately with participants details. This runs throughout the findings section and authors should pay critical attention to it.

**Response:** Thank you for your thoughtful comment. We now provide in-depth participant profiles for each participant in Supplement Table 1. We note this in the text on page 13.

Sincerely,

The Authors
