# Supplementary material for: Systemic effects of the COVID pandemic on rural black American men’s interpersonal relationships: A phenomenological examination
Source: PLoS One. 2024 Apr 17;19(4):e0297876. doi: 10.1371/journal.pone.0297876 (PMC11023195; doi:10.1371/journal.pone.0297876)
Supplement: Peer review history

# Peer review: Decision Letter 0

**DOI:** 10.1371/journal.pone.0297876.r001

**Authors:** Ejercito Mangawa Balay-odao

7 Dec 2023

PONE-D-23-31672Systemic Effects of the COVID Pandemic on Rural Black American Men’s Interpersonal Relationships: A Phenomenological ExaminationPLOS ONE

Dear Dr. Curtis,

Thank you for submitting your manuscript to PLOS ONE. After careful consideration, we feel that it has merit but does not fully meet PLOS ONE’s publication criteria as it currently stands. Therefore, we invite you to submit a revised version of the manuscript that addresses the points raised during the review process.

Please submit your revised manuscript by Jan 21 2024 11:59PM. If you will need more time than this to complete your revisions, please reply to this message or contact the journal office at plosone@plos.org. When you're ready to submit your revision, log on to https://www.editorialmanager.com/pone/ and select the 'Submissions Needing Revision' folder to locate your manuscript file.

Please include the following items when submitting your revised manuscript:

- A rebuttal letter that responds to each point raised by the academic editor and reviewer(s). You should upload this letter as a separate file labeled 'Response to Reviewers'.
- A marked-up copy of your manuscript that highlights changes made to the original version. You should upload this as a separate file labeled 'Revised Manuscript with Track Changes'.
- An unmarked version of your revised paper without tracked changes. You should upload this as a separate file labeled 'Manuscript'.

If you would like to make changes to your financial disclosure, please include your updated statement in your cover letter. Guidelines for resubmitting your figure files are available below the reviewer comments at the end of this letter.

If applicable, we recommend that you deposit your laboratory protocols in protocols.io to enhance the reproducibility of your results. Protocols.io assigns your protocol its own identifier (DOI) so that it can be cited independently in the future. For instructions see: https://journals.plos.org/plosone/s/submission-guidelines#loc-laboratory-protocols. Additionally, PLOS ONE offers an option for publishing peer-reviewed Lab Protocol articles, which describe protocols hosted on protocols.io. Read more information on sharing protocols at https://plos.org/protocols?utm_medium=editorial-email&utm_source=authorletters&utm_campaign=protocols.

We look forward to receiving your revised manuscript.

Kind regards,

Ejercito Mangawa Balay-odao, PhD

Academic Editor

PLOS ONE

Journal requirements:

When submitting your revision, we need you to address these additional requirements.

1. Please ensure that your manuscript meets PLOS ONE's style requirements, including those for file naming. The PLOS ONE style templates can be found at

https://journals.plos.org/plosone/s/file?id=wjVg/PLOSOne_formatting_sample_main_body.pdf and

https://journals.plos.org/plosone/s/file?id=ba62/PLOSOne_formatting_sample_title_authors_affiliations.pdf

2. We note that the grant information you provided in the ‘Funding Information’ and ‘Financial Disclosure’ sections do not match.

When you resubmit, please ensure that you provide the correct grant numbers for the awards you received for your study in the ‘Funding Information’ section.

3. Thank you for stating the following financial disclosure:

“This work was in part supported by the National Institute on Drug Abuse under Grant R01DA029488, the National Institute on Alcohol Abuse and Alcoholism under Grant R01AA026623, and the National Center for Advancing Translational Sciences of the National Institutes of Health under Award Numbers UL1TR002378 and TL1TR002382.”

Please state what role the funders took in the study.  If the funders had no role, please state: "The funders had no role in study design, data collection and analysis, decision to publish, or preparation of the manuscript."

If this statement is not correct you must amend it as needed.

Please include this amended Role of Funder statement in your cover letter; we will change the online submission form on your behalf.

4. We note that you have indicated that data from this study are available upon request. PLOS only allows data to be available upon request if there are legal or ethical restrictions on sharing data publicly. For more information on unacceptable data access restrictions, please see http://journals.plos.org/plosone/s/data-availability#loc-unacceptable-data-access-restrictions.

In your revised cover letter, please address the following prompts:

a) If there are ethical or legal restrictions on sharing a de-identified data set, please explain them in detail (e.g., data contain potentially sensitive information, data are owned by a third-party organization, etc.) and who has imposed them (e.g., an ethics committee). Please also provide contact information for a data access committee, ethics committee, or other institutional body to which data requests may be sent.

b) If there are no restrictions, please upload the minimal anonymized data set necessary to replicate your study findings as either Supporting Information files or to a stable, public repository and provide us with the relevant URLs, DOIs, or accession numbers. For a list of acceptable repositories, please see http://journals.plos.org/plosone/s/data-availability#loc-recommended-repositories.

We will update your Data Availability statement on your behalf to reflect the information you provide.

5. Please include captions for your Supporting Information files at the end of your manuscript, and update any in-text citations to match accordingly. Please see our Supporting Information guidelines for more information: http://journals.plos.org/plosone/s/supporting-information.

6. Please review your reference list to ensure that it is complete and correct. If you have cited papers that have been retracted, please include the rationale for doing so in the manuscript text, or remove these references and replace them with relevant current references. Any changes to the reference list should be mentioned in the rebuttal letter that accompanies your revised manuscript. If you need to cite a retracted article, indicate the article’s retracted status in the References list and also include a citation and full reference for the retraction notice.

Additional Editor Comments:

The author/s did not mentioned if they use equator checklist. Please check the article according to the COREQ checklist. (https://www.equator-network.org/reporting-guidelines/coreq/)

Reviewers' comments:

Reviewer's Responses to Questions

**Comments to the Author**

1. Is the manuscript technically sound, and do the data support the conclusions?

The manuscript must describe a technically sound piece of scientific research with data that supports the conclusions. Experiments must have been conducted rigorously, with appropriate controls, replication, and sample sizes. The conclusions must be drawn appropriately based on the data presented. 

Reviewer #1: Partly

Reviewer #2: Partly

Reviewer #3: Yes

********** 

2. Has the statistical analysis been performed appropriately and rigorously? 

Reviewer #1: Yes

Reviewer #2: N/A

Reviewer #3: Yes

********** 

3. Have the authors made all data underlying the findings in their manuscript fully available?

The PLOS Data policy requires authors to make all data underlying the findings described in their manuscript fully available without restriction, with rare exception (please refer to the Data Availability Statement in the manuscript PDF file). The data should be provided as part of the manuscript or its supporting information, or deposited to a public repository. For example, in addition to summary statistics, the data points behind means, medians and variance measures should be available. If there are restrictions on publicly sharing data—e.g. participant privacy or use of data from a third party—those must be specified.

Reviewer #1: Yes

Reviewer #2: Yes

Reviewer #3: Yes

********** 

4. Is the manuscript presented in an intelligible fashion and written in standard English?

PLOS ONE does not copyedit accepted manuscripts, so the language in submitted articles must be clear, correct, and unambiguous. Any typographical or grammatical errors should be corrected at revision, so please note any specific errors here.

Reviewer #1: No

Reviewer #2: Yes

Reviewer #3: Yes

********** 

5. Review Comments to the Author

Please use the space provided to explain your answers to the questions above. You may also include additional comments for the author, including concerns about dual publication, research ethics, or publication ethics. (Please upload your review as an attachment if it exceeds 20,000 characters)

Reviewer #1: The chosen theoretical frameworks were well justified to inform your research approach. Data analysis was described adequately.

It is unclear the study timeline with the description of "The COVID-19 pandemic occurred after Wave 4 of data collection was completed." Does this mean your study occurred before COVID-19? The explanations for the different waves were confusing. It appears to me that only waves 1 and 2 were included in this study, and that AMP includes all 6 waves. Please provide some further explanation of the waves of data collection in your methods section and what the relavence of the different waves are to this particular study. What were the purposes of each wave, and why did interviews occur during various waves of your study? Was Table 1: demographic information collected during interview or on a survey form? This is not described. Assuming this is done on a survey format since there was missing data in the response.

There are some abbreviations not abbreviated correctly (e.g. CDC, page 31; U.S. was abbreviated then spelt full form in Strengths & limitations, page 37). Typos in the in text citation (e.g. Robins et al., 2023, page 9). Contractions noted (e.g. "they're", pages 31 & 32).

Some errors in the reference list (e.g. Allen, Q 2016, journal name and issue were not italic. Some links to references were not provided (e.g. Hardy & Logan 2021 https://www.brookings.edu/articles/the-way-back-assessing-economic-recovery-among-black-americans-during-covid-19/).

Reviewer #2: The essential sections of a qualitative paper were presented.

In the background, the author provided sufficient information to establish the research gap fully. However, aside from the theoretical frameworks presented, it would have been better if the author included the philosophical underpinnings, which are particularly important in qualitative research because they influence how researchers approach their research questions, collect and analyze data, and interpret their findings.

In the methods section, the author wisely used a qualitative approach in this study as it allowed for a rich and nuanced understanding of the experiences of Black American men. Moreover, information about the participants and data collection and analysis were included. However, I am concerned with using a hermeneutic phenomenological qualitative approach since there is a potential for subjectivity. In hermeneutic phenomenology, the researcher is an active participant in the research process. Hence, the researcher’s own experiences and background might have influenced his interpretation of the data (e.g., Queer, Black American man who grew up in a low-resource environment; has made me keenly sensitive to the systems of oppression; I was, and remain, deeply connected to the men’s lived experiences highlighted in this study). While the author mentioned under the subheading trustworthiness about having a reflexive attitude and maintaining a keen awareness of the researcher’s biases and preconceived beliefs, it is unclear how he specifically did this throughout the data-gathering process. It is unclear how reflexivity and bracketing were done.

In the findings and discussion section, the author did a good job presenting relevant information that suggests that the pandemic has exacerbated existing social, economic, and health inequalities for Black American men and that these inequalities have also had a negative impact on their interpersonal relationships. It was also presented that the pandemic had a mixed impact on Black American men’s relationships, with some positive and negative outcomes.

The findings supported the conclusion. The author highlighted the importance of considering the role of interpersonal relationships in the health and well-being of Black American men. Additionally, the author reiterated the need for stakeholders to consider men’s relational health when developing and implementing pandemic recovery efforts.

Overall, this study provides a valuable contribution to our understanding of the impact of the COVID-19 pandemic on Black American men. In particular, the study provides valuable insights into the experiences of rural Black American men during the COVID-19 pandemic, highlighting the importance of interpersonal relationships for their mental and emotional well-being. The study’s conclusions are significant and have implications for future research and practice; thus, it is recommended for publication.

Reviewer #3: 1. Misarrangement of Key words: Please ensure Key words are arranged in an alphabetical order.

2. At line three (3) of the abstract, U.S should not be abbreviated Sub-themes numbering: Please provide full meaning to U.S instead of the abbreviation in the Abstract. Also remove the numbers attached to the sub-themes at the abstract

3. At the abstract section, line 5, this study aimed to “qualitatively explore the effects”: Please remove the “qualitatively” and maintain the “explore”

4. No recommendations provided at the abstract: Please state at least two recommendations (be specific) in the abstract section rather than presenting it as “discussed”

5. Participants biodata missing: Authors should provide a presentation of biodata of study participants at the finding’s column.

6. At the findings section, direct quotations were not supported with participants details: Authors to take critical look at the direct quotations at the findings section and support quotes appropriately with participants details. This runs throughout the findings section and authors should pay critical attention to it..

********** 

6. PLOS authors have the option to publish the peer review history of their article (what does this mean?). If published, this will include your full peer review and any attached files.

If you choose “no”, your identity will remain anonymous but your review may still be made public.

**Do you want your identity to be public for this peer review?** For information about this choice, including consent withdrawal, please see our Privacy Policy.

Reviewer #1: **Yes: **Thomas Aaron Ricks

Reviewer #2: No

Reviewer #3: No

**********

[NOTE: If reviewer comments were submitted as an attachment file, they will be attached to this email and accessible via the submission site. Please log into your account, locate the manuscript record, and check for the action link "View Attachments". If this link does not appear, there are no attachment files.]

While revising your submission, please upload your figure files to the Preflight Analysis and Conversion Engine (PACE) digital diagnostic tool, https://pacev2.apexcovantage.com/. PACE helps ensure that figures meet PLOS requirements. To use PACE, you must first register as a user. Registration is free. Then, login and navigate to the UPLOAD tab, where you will find detailed instructions on how to use the tool. If you encounter any issues or have any questions when using PACE, please email PLOS at figures@plos.org. Please note that Supporting Information files do not need this step.

Attachment Submitted filename: Review Results Dec2023.docx

# Author comment: Author response to Decision Letter 0

**DOI:** 10.1371/journal.pone.0297876.r002

23 Dec 2023

Dear Dr. Editors & Reviewers,

Thank you for the excellent news that we have been invited to resubmit our manuscript, “Systemic Effects of the COVID-19 Pandemic on Rural Black American Men’s Interpersonal Relationships: A Phenomenological Examination” for exclusive consideration in the PLOS One. Below, we detail our revisions and responses, in bold, to each concern the reviewers raised.

Reviewer 1

Comment: The chosen theoretical frameworks were well justified to inform your research approach. Data analysis was described adequately. It is unclear the study timeline with the description of "The COVID-19 pandemic occurred after Wave 4 of data collection was completed." Does this mean your study occurred before COVID-19? The explanations for the different waves were confusing. It appears to me that only waves 1 and 2 were included in this study, and that AMP includes all 6 waves. Please provide some further explanation of the waves of data collection in your methods section and what the relevance of the different waves are to this particular study. What were the purposes of each wave, and why did interviews occur during various waves of your study? 

Response: We have revised the Participants section to add additional context concerning the larger quantitative study within which the current quantitative study took place. We also clarified the timeline concerning when waves 1 and 2 of interviews took place. This section now reads:

The COVID-19 pandemic occurred after Wave 4 of quantitative data collection was completed. The first wave of interviews was conducted from April 2020 to December 2020, while the second wave was conducted from September 2021 to October 2021.

Comment: Was Table 1: demographic information collected during interview or on a survey form? This is not described. Assuming this is done on a survey format since there was missing data in the response.

Response: That is correct, demographic information was calculated using survey data from Wave 4 of quantitative data collection. We have clarified this language on page 11. It now reads as:

The COVID-19 pandemic occurred after Wave 4 of quantitative data collection was completed (i.e. January 2020). The first wave of interviews was conducted from April 2020 to December 2020, while the second wave was conducted from September 2021 to October 2021.

Comment: There are some abbreviations not abbreviated correctly (e.g. CDC, page 31; U.S. was abbreviated then spelt full form in Strengths & limitations, page 37). Typos in the in text citation (e.g. Robins et al., 2023, page 9). Contractions noted (e.g. "they're", pages 31 & 32). Some errors in the reference list (e.g. Allen, Q 2016, journal name and issue were not italic. Some links to references were not provided (e.g. Hardy & Logan 2021 https://www.brookings.edu/articles/the-way-back-assessing-economic-recovery-among-black-americans-during-covid-19/).

Response: We have thoroughly reviewed the manuscript and references section to correct these errors and have corrected any found mistakes.

Reviewer 2

Comment: In the background, the author provided sufficient information to establish the research gap fully. However, aside from the theoretical frameworks presented, it would have been better if the author included the philosophical underpinnings, which are particularly important in qualitative research because they influence how researchers approach their research questions, collect and analyze data, and interpret their findings.

Response: We have added a new section entitled “Paradigmatic Orientation” that articulates that critical post-modernism and social constructionism served as guiding philosophical paradigms that guided the development, and implementation of the current study (see pages 6-8). 

Comment: In the methods section, the author wisely used a qualitative approach in this study as it allowed for a rich and nuanced understanding of the experiences of Black American men. Moreover, information about the participants and data collection and analysis were included. However, I am concerned with using a hermeneutic phenomenological qualitative approach since there is a potential for subjectivity. In hermeneutic phenomenology, the researcher is an active participant in the research process. Hence, the researcher’s own experiences and background might have influenced his interpretation of the data (e.g., Queer, Black American man who grew up in a low-resource environment; has made me keenly sensitive to the systems of oppression; I was, and remain, deeply connected to the men’s lived experiences highlighted in this study). While the author mentioned under the subheading trustworthiness about having a reflexive attitude and maintaining a keen awareness of the researcher’s biases and preconceived beliefs, it is unclear how he specifically did this throughout the data-gathering process. It is unclear how reflexivity and bracketing were done.

Response: Thank you for your insightful comment. While bracketing is a central component to many qualitative approaches it was purposely not integrated in this study or analysis. As articulated by Madison and the tenets of critical ethnography, the notion that a researcher can separate their lived experiences from that of their participants is unreasonable at it would (a) ignore my (MGC) status as an insider within the Black American and male-presenting communities and (b). Instead, I leveraged my status to create rapport with participants and cultivate and maintain a sensitivity to the power positions, choices, and effects that my lived experiences had on the research process. This sensitivity was documented via consistent memo writing throughout the data collection and analysis process. Externals audits by secondary researchers were utilized to ensure that these dynamics did not negatively influence the research process. We outline this process on page 18.

Reviewer 3

Comment: 2. At line three (3) of the abstract, U.S should not be abbreviated Sub-themes numbering: Please provide full meaning to U.S instead of the abbreviation in the Abstract. Also remove the numbers attached to the sub-themes at the abstract

Response: This has been corrected.

Comment: 3. At the abstract section, line 5, this study aimed to “qualitatively explore the effects”: Please remove the “qualitatively” and maintain the “explore”

Response: This has been corrected.

Comment: 4. No recommendations provided at the abstract: Please state at least two recommendations (be specific) in the abstract section rather than presenting it as “discussed”

Response: We added two recommendations. The final sentence in the abstract now reads:

Future research should be dedicated to (1) investigating the effects of the COVID-19 pandemic on fathers, as prior research has nearly exclusively focused on mothers' experiences and (2) delineating protective effects of rural Black American men’s involvement in the Black Church from their individual spiritualities to gain a more comprehensive understanding of the influence of contextual crisis on their long-term health and wellbeing.

Comment: 5. Participants biodata missing: Authors should provide a presentation of biodata of study participants at the finding’s column.

Response: No biological data was collected was collected in this study so there was no biological data to report. 

Comment: 6. At the findings section, direct quotations were not supported with participants details: Authors to take critical look at the direct quotations at the findings section and support quotes appropriately with participants details. This runs throughout the findings section and authors should pay critical attention to it.

Response: Thank you for your thoughtful comment. We now provide in-depth participant profiles for each participant in Supplement Table 1. We note this in the text on page 13.

Sincerely,

The Authors

Attachment Submitted filename: Revision Letter 1.docx

# Peer review: Decision Letter 1

**DOI:** 10.1371/journal.pone.0297876.r003

**Authors:** Ejercito Mangawa Balay-odao

15 Jan 2024

Systemic Effects of the COVID Pandemic on Rural Black American Men’s Interpersonal Relationships: A Phenomenological Examination

PONE-D-23-31672R1

Dear Dr. Michael Curtis,

We’re pleased to inform you that your manuscript has been judged scientifically suitable for publication and will be formally accepted for publication once it meets all outstanding technical requirements.

Within one week, you’ll receive an e-mail detailing the required amendments. When these have been addressed, you’ll receive a formal acceptance letter and your manuscript will be scheduled for publication.

An invoice for payment will follow shortly after the formal acceptance. To ensure an efficient process, please log into Editorial Manager at http://www.editorialmanager.com/pone/, click the 'Update My Information' link at the top of the page, and double check that your user information is up-to-date. If you have any billing related questions, please contact our Author Billing department directly at authorbilling@plos.org.

If your institution or institutions have a press office, please notify them about your upcoming paper to help maximize its impact. If they’ll be preparing press materials, please inform our press team as soon as possible -- no later than 48 hours after receiving the formal acceptance. Your manuscript will remain under strict press embargo until 2 pm Eastern Time on the date of publication. For more information, please contact onepress@plos.org.

Kind regards,

Ejercito Mangawa Balay-odao, PhD

Academic Editor

PLOS ONE

Additional Editor Comments (optional):

Reviewers' comments:

Reviewer's Responses to Questions

**Comments to the Author**

1. If the authors have adequately addressed your comments raised in a previous round of review and you feel that this manuscript is now acceptable for publication, you may indicate that here to bypass the “Comments to the Author” section, enter your conflict of interest statement in the “Confidential to Editor” section, and submit your "Accept" recommendation.

Reviewer #1: All comments have been addressed

Reviewer #3: All comments have been addressed

**********

2. Is the manuscript technically sound, and do the data support the conclusions?

The manuscript must describe a technically sound piece of scientific research with data that supports the conclusions. Experiments must have been conducted rigorously, with appropriate controls, replication, and sample sizes. The conclusions must be drawn appropriately based on the data presented. 

Reviewer #1: Yes

Reviewer #3: Yes

**********

3. Has the statistical analysis been performed appropriately and rigorously? 

Reviewer #1: Yes

Reviewer #3: Yes

**********

4. Have the authors made all data underlying the findings in their manuscript fully available?

The PLOS Data policy requires authors to make all data underlying the findings described in their manuscript fully available without restriction, with rare exception (please refer to the Data Availability Statement in the manuscript PDF file). The data should be provided as part of the manuscript or its supporting information, or deposited to a public repository. For example, in addition to summary statistics, the data points behind means, medians and variance measures should be available. If there are restrictions on publicly sharing data—e.g. participant privacy or use of data from a third party—those must be specified.

Reviewer #1: Yes

Reviewer #3: Yes

**********

5. Is the manuscript presented in an intelligible fashion and written in standard English?

PLOS ONE does not copyedit accepted manuscripts, so the language in submitted articles must be clear, correct, and unambiguous. Any typographical or grammatical errors should be corrected at revision, so please note any specific errors here.

Reviewer #1: Yes

Reviewer #3: Yes

**********

6. Review Comments to the Author

Please use the space provided to explain your answers to the questions above. You may also include additional comments for the author, including concerns about dual publication, research ethics, or publication ethics. (Please upload your review as an attachment if it exceeds 20,000 characters)

Reviewer #1: The authors have address all the concerns that I have mentioned in the previous review. The author has also considered the revision well and attended to other reviewers comments. I am satisfied with the quality of this paper and happy to recommend for an acceptance for publication.

Reviewer #3: (No Response)

**********

7. PLOS authors have the option to publish the peer review history of their article (what does this mean?). If published, this will include your full peer review and any attached files.

If you choose “no”, your identity will remain anonymous but your review may still be made public.

**Do you want your identity to be public for this peer review?** For information about this choice, including consent withdrawal, please see our Privacy Policy.

Reviewer #1: **Yes: **Thomas Aaron Ricks

Reviewer #3: No

**********

# Editor report: Acceptance letter

**DOI:** 10.1371/journal.pone.0297876.r004

**Authors:** Ejercito Mangawa Balay-odao

25 Jan 2024

PONE-D-23-31672R1 

PLOS ONE

Dear Dr. Curtis, 

I'm pleased to inform you that your manuscript has been deemed suitable for publication in PLOS ONE. Congratulations! Your manuscript is now being handed over to our production team.

At this stage, our production department will prepare your paper for publication. This includes ensuring the following:

* All references, tables, and figures are properly cited

* All relevant supporting information is included in the manuscript submission,

* There are no issues that prevent the paper from being properly typeset

If revisions are needed, the production department will contact you directly to resolve them. If no revisions are needed, you will receive an email when the publication date has been set. At this time, we do not offer pre-publication proofs to authors during production of the accepted work. Please keep in mind that we are working through a large volume of accepted articles, so please give us a few weeks to review your paper and let you know the next and final steps. 

Lastly, if your institution or institutions have a press office, please let them know about your upcoming paper now to help maximize its impact. If they'll be preparing press materials, please inform our press team within the next 48 hours. Your manuscript will remain under strict press embargo until 2 pm Eastern Time on the date of publication. For more information, please contact onepress@plos.org.

If we can help with anything else, please email us at customercare@plos.org.

Thank you for submitting your work to PLOS ONE and supporting open access. 

Kind regards, 

PLOS ONE Editorial Office Staff

on behalf of

Dr. Ejercito Mangawa Balay-odao 

Academic Editor

PLOS ONE
